# Glycosylation limits forward trafficking of the tetraspan membrane protein PMP22

**DOI:** 10.1016/j.jbc.2021.100719

**Published:** 2021-04-30

**Authors:** Justin T. Marinko, Madison T. Wright, Jonathan P. Schlebach, Katherine R. Clowes, Darren R. Heintzman, Lars Plate, Charles R. Sanders

**Affiliations:** 1 Department of Biochemistry, Vanderbilt University, Nashville, Tennessee, USA; 2 Center for Structural Biology, Vanderbilt University, Nashville, Tennessee, USA; 3 Department of Chemistry, Vanderbilt University, Nashville, Tennessee, USA; 4 Department of Chemistry, Indiana University, Bloomington, Indiana, USA; 5 Department of Pathology, Microbiology, and Immunology, Vanderbilt University, Nashville, Tennessee, USA; 6 Department of Biological Sciences, Vanderbilt University, Nashville, Tennessee, USA

**Keywords:** Charcot–Marie–Tooth disease (CMTD), peripheral neuropathy, peripheral myelin protein 22 (PMP22), ER quality control, N-linked glycosylation, trafficking, CMTD, Charcot–Marie–Tooth disease, CNX, calnexin, co-IP, coimmunoprecipitation, DMEM, Dulbecco’s modified Eagle medium, EMC, ER membrane protein complex, ER, endoplasmic reticulum, ERAD, ER-associated degradation, ERQC, ER quality control network, FBS, fetal bovine serum, GO, gene ontology, gRNA, guide RNA, OST, oligosaccharyltransferase, PM, plasma membrane, PMP22, peripheral myelin protein 22, PNS, peripheral nervous system, RER1, retention in ER sorting receptor 1, RSC, rat Schwann cell, TM, transmembrane, TMT, tandem mass tag

## Abstract

Peripheral myelin protein 22 (PMP22) folds and trafficks inefficiently, with only 20% of newly expressed protein trafficking to the cell surface. This behavior is exacerbated in many of the mutants associated with Charcot–Marie–Tooth disease, motivating further study. Here we characterized the role of N-glycosylation in limiting PMP22 trafficking. We first eliminated N-glycosylation using an N41Q mutation, which resulted in an almost 3-fold increase in trafficking efficiency of wildtype (WT) PMP22 and a 10-fold increase for the severely unstable L16P disease mutant in HEK293 cells, with similar results in Schwann cells. Total cellular levels were also much higher for the WT/N41Q mutant, although not for the L16P/N41Q form. Depletion of oligosaccharyltransferase OST-A and OST-B subunits revealed that WT PMP22 is N-glycosylated posttranslationally by OST-B, whereas L16P is cotranslationally glycosylated by OST-A. Quantitative proteomic screens revealed similarities and differences in the interactome for WT, glycosylation-deficient, and unstable mutant forms of PMP22 and also suggested that L16P is sequestered at earlier stages of endoplasmic reticulum quality control. CRISPR knockout studies revealed a role for retention in endoplasmic reticulum sorting receptor 1 (RER1) in limiting the trafficking of all three forms, for UDP-glucose glycoprotein glucosyltransferase 1 (UGGT1) in limiting the trafficking of WT and L16P but not N41Q, and calnexin (CNX) in limiting the trafficking of WT and N41Q but not L16P. This work shows that N-glycosylation is a limiting factor to forward trafficking PMP22 and sheds light on the proteins involved in its quality control.

Secreted and transmembrane proteins comprise over one-third of the human proteome, passing through the endoplasmic reticulum (ER) en route to their ultimate destinations (1, 2, 3). For most integral membrane proteins, integration into the ER membrane is intimately coupled to translation. Translating ribosomes associate with the Sec61–translocon complex to thread transmembrane (TM) segments through a central water-exposed pore (4, 5, 6). This pore contains a lateral gate that allows translocating polypeptides to sample both lipid and hydrophilic environments (7). Soluble proteins translocate through the pore to enter the ER lumen, while TM helices partition laterally into the ER membrane. Most membrane proteins adopt their secondary structure and attain correct membrane topology at this initial stage of assembly. Once fully synthesized, proteins are released from the translocon, diffuse away, and complete the second stage of membrane protein folding: formation of tertiary and quaternary structure (1, 8). Once properly folded, proteins traffic beyond the ER *via* exit sites to the Golgi complex and from there to their destination membrane (2). Proteins that fail to adopt proper structure are retained in the ER to provide additional time for folding or, failing that, are targeted for elimination, most often by ER-associated degradation (ERAD) or ER-associated autophagy (9).

Protein folding in the ER is under constant surveillance by the resident ER quality control network (ERQC) (1, 10, 11), which contains numerous folding sensors, chaperones, and other proteins, including those involved in ERAD and ER-associated autophagy. Collectively, these proteins monitor, assist, and execute logic decisions as to whether to retain, degrade, or authorize exit of nascent proteins from the ER. Much is known about the molecular details of this pathway for soluble proteins, but less is understood for TM proteins. Here, we seek to illuminate how ERQC manages quality control decisions for human peripheral myelin protein 22 (PMP22).

PMP22 is a tetraspan integral membrane protein (Fig. 1*A*) that is highly expressed in the plasma membrane (PM) of myelinating Schwann cells in the peripheral nervous system (PNS) (12, 13). The specific functions of PMP22 are not well understood (14, 15, 16) but include a structural role in both the maintenance and development of compact myelin (17). PMP22 shares ∼60% sequence similarity with claudin-15, one of the structural proteins involved in maintaining tight junctions (18). Moreover, when reconstituted into liposomes, PMP22 can induce flattening and wrapping of the vesicles to form myelin-like assemblies (19). Mutations in the *pmp22* gene, including gene duplication, gene deletion, or any one of more than 40 known single nucleotide polymorphisms, cause a range of progressive peripheral neuropathies including Charcot–Marie–Tooth disease types 1A and E, hereditary neuropathy with liability to pressure palsies, and Dejerine–Sottas syndrome (17, 20). For the sake of simplicity, we collectively refer to these peripheral neuropathies as Charcot–Marie–Tooth disease (CMTD), which together afflict ∼1 in 2500 individuals, with 70% of cases being due to mutations that impact *pmp22* (17). The underlying cause of the disease is dysmyelination of PNS nerves, which reduces nerve conduction velocity along the peripheral axons. Depending on the causative mutation, CMTD ranges in severity, with symptoms including abnormalities of peripheral axons, impaired tendon reflexes, progressive weakness of distal musculature, muscle cramping, and abnormal gait. Patients with a severe phenotype can be confined to a wheelchair, experience chronic pain, and/or be afflicted with blindness and loss of hearing (21, 22, 23). There is presently no treatment for CMTD beyond symptom management (22).

**Figure 1:**
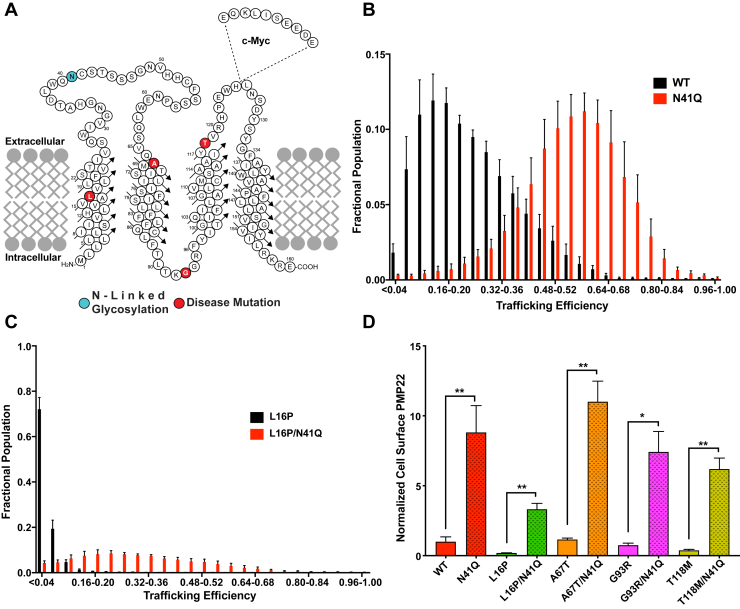
**N-glycosylation limits forward trafficking of PMP22.***A*, topology diagram of PMP22 in a membrane. The sequential location of the c-myc tag used in the trafficking assay is shown. Disease variant sites for mutants examined in this study are highlighted in *red*, and the site of N-linked glycosylation is shown in *cyan*. *B*, population distribution of PMP22 trafficking efficiencies measured in individual HEK293 cells for WT (*black*) and N41Q (*red*) and for (*C*) L16P (*black*) and L16P/N41Q (*red*). Measurements reflect the results from five biological replicates with 2500 cells measured per replicate. Error bars represent standard deviations (SD) of the replicates. *D*, normalized cell surface expression of PMP22 variants and their glycosylation-deficient counterpart. Values reflect outcomes from five biological replicates with 2500 cells measured per replicate. All values were normalized to WT PMP22 cell surface expression based on results from paired biological replicates. Error bars represent SD of the replicates. Student’s *t* test was used for statistical analysis. ∗*p* < 0.05, ∗∗*p* < 0.01.

The most common form of CMTD (type 1A) is caused by overproduction of PMP22 due to a heterozygous duplication of chromosome fragment 17p.11-2.12, resulting in trisomy and three copies of the *pmp22* gene (20). One hypothesis for how WT PMP22 overexpression causes disease is that increased production of the protein results in oversaturation of ERQC, leading to accumulation of misfolded protein and resulting toxicity and/or cell stress (24, 25). This seems especially plausible in light of data indicating that even under healthy conditions, PMP22 is prone to misfold, with only 20% of newly expressed protein trafficking to the cell surface (24, 26, 27, 28). Mutant forms of PMP22 are known to traffic even less efficiently than WT, consistent with the notion that peripheral neuropathies associated with these PMP22 variants also are the consequence of defects in PMP22 trafficking.

We have previously carried out studies to elucidate the molecular defects in PMP22 that cause it to be mistrafficking-prone. Biophysical studies of PMP22 revealed that the WT protein is only moderately stable, with the folded conformation favored over unfolded protein by only 1.5 ± 0.1 kcal mol^−1^ in detergent micelles (27, 29). Although WT PMP22 is undoubtably more stable in native membranes, marginal stability of WT PMP22 likely is closely related for the known inefficient folding and trafficking to the PM. Disease mutant forms of PMP22 were shown to be even less stable than WT. Indeed, quantitative cell trafficking measurements for this same panel of mutants revealed linear relationships between PMP22 surface trafficking efficiency and patient nerve conduction velocities, between PMP22 stability and surface trafficking efficiencies, and between PMP22 stability and patient nerve conduction velocities (27). PMP22-linked CMTD appears to be a disease that resembles cystic fibrosis in the sense that the vast majority of disease cases are caused by folding defects and mistrafficking (1, 30). ERQC is evidently attuned to be able to assess the conformational stability of PMP22.

Exactly how PMP22 folding is monitored and managed in the ER is not well understood. Common ER-resident chaperones involved in the folding of many soluble proteins, such as the heat shock protein 70 binding immunoglobin factor, calreticulin, or the thiol-isomerase ERp57, do not appear to be important for the maturation of PMP22 (31, 32, 33). On the other hand, the lectin chaperone calnexin (CNX) has been shown to engage WT and disease variants of PMP22 (31, 32, 33, 34, 35). Moreover, data indicate that retention in ER sorting receptor 1 (RER1) can retrieve disease variants of PMP22 from the Golgi complex and return it to the ER (35). Beyond this, little is understood about how the components of ERQC that engage nascent PMP22 determine the balance between its forward trafficking, retention in the ER, and targeting for degradation. In this paper, we show that N-linked glycosylation of PMP22 significantly hinders the forward trafficking of both WT and disease variants of the protein. We also explored how PMP22 is N-glycosylated by the oligosaccharyltransferases A and B (OST-A, OST-B). Furthermore, quantitative proteomics and related pathway analysis was used to identify how the assembly of various forms of PMP22 (WT, nonglycosylated, and disease mutant forms) is differentially managed by ERQC. Finally, we used CRISPR-Cas9 to generate knockout cell lines to illuminate the roles of several key ERQC proteins in mediating the trafficking of different forms of PMP22.

## Results

### N-glycosylation limits forward trafficking of PMP22

PMP22, like most proteins that are inserted into the ER, is posttranslationally modified by addition of a 14-sugar complex oligosaccharide (N-glycan) (36, 37). This N-glycan is added to asparagine residues associated with the sequence motif N-X-S/T (where X is any amino acid except proline). PMP22 contains a single N-glycosylation site, located in its extracellular loop 1 (ECL1) at asparagine 41 (N41; Fig. 1*A* cyan). Within the lumen of the ER, changing the identity of the N-glycan through addition or removal of monosaccharides can trigger binding of client proteins by the folding-assistive lectin chaperones (37, 38). Previous work has shown that for PMP22 the N-glycan may play a modest role in oligomer stability (39) but does not seem to affect protein function. We have also recently shown that N-glycosylation of PMP22 does not affect its distinct membrane phase preference for cholesterol-rich ordered phase domains (40). Here we tested the impact on human PMP22 trafficking of mutating N41 to a glutamine (N41Q), thereby rendering PMP22 glycosylation deficient.

Using single-cell flow cytometry–based assays to directly quantitate both cell surface and internal (mistrafficked) levels of PMP22 in individual cells, we measured the trafficking efficiencies (the amount of cell surface PMP22 over total expressed PMP22) for the WT *versus* N41Q mutant forms PMP22 in HEK293 cells (Fig. 1*B*). WT PMP22 displayed a trafficking efficiency of 19 ± 5% (mean ± standard deviation; Fig. 1*B*, black), which corroborates with previous values obtained using the same assay with Madin–Darby Canine Kidney cells and also previous measurements using sciatic nerve lysates (24, 26, 27). Remarkably, it was seen that N41Q PMP22 trafficked to the surface of HEK293 cells with a nearly 3-fold greater surface trafficking efficiency of 53 ± 7% (Fig. 1*B*, red).

We next examined a highly destabilized disease-causing variant of PMP22, L16P, to see if glycosylation also affected its trafficking (Fig. 1*C*). This severe CMTD mutation in PMP22 causes a kink in TM1 that destabilizes the protein and causes the majority of the protein to be retained intracellularly (27, 41, 42, 43). Our experiments reflect these previous observations, as L16P PMP22 was seen to surface-traffic with only 3 ± 1% efficiency (Fig. 1*C*, black). However, when the glycosylation site was removed (L16P/N41Q) the trafficking efficiency increased more than 10-fold to 34 ± 10%, (Fig. 1*C*, red). We also observed a pronounced increase in surface trafficking upon removal of N-linked glycosylation for three additional CMTD mutant forms: one known to have WT-like stability (A67T) and two with stability intermediate between WT and the very unstable L16P (G93R and T118M) (Figs. 1*D* and S1) (27). Finally, our trafficking assay also provided the internal (Fig. S2*A*) and total (Fig. S2*B*) expression levels for WT and each mutant in HEK293 cells. For WT, A67T, G93R, and T118M forms of PMP22, elimination of glycosylation resulted in a pronounced and statistically significant increase in the total cellular PMP22. For the L16P mutant, only a statistically insignificant increase in total protein level was seen upon elimination of glycosylation.

**Figure 2:**
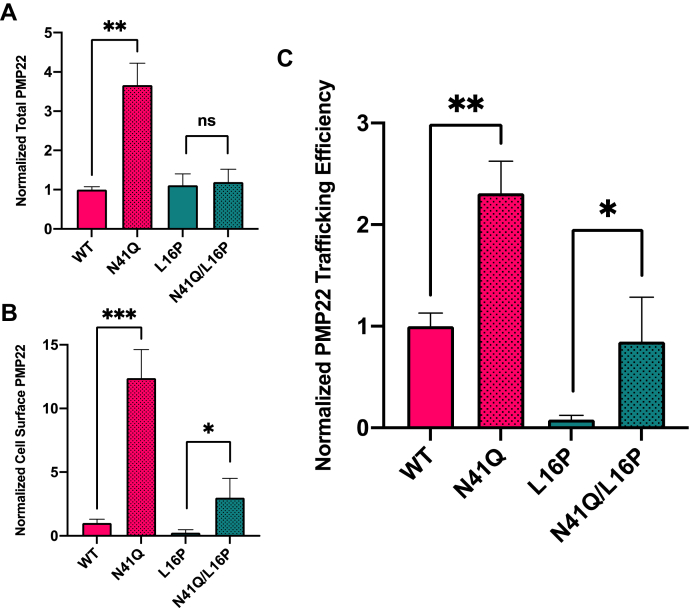
**PMP22 trafficking in rat Schwann cells**. Normalized (relative to WT) (*A*) total and (*B*) cell surface expression of WT and N41Q PMP22N41Q, as well as L16P and N41Q/L16P PMP22 variants. *C*, normalized trafficking efficiency of WT PMP22 and variants. Values reflect averages from three biological replicates in which 250 to 900 cells were measured per replicate. All values were normalized to WT PMP22 data collected in paired biological replicates. WT values in each replicate were normalized to the average WT values to show variability between replicates. Error bars represent standard deviation of the replicate averages. Student’s *t* test was used for statistical analysis. ns, not significant; ∗*p* < 0.05, ∗∗*p* < 0.01.

We sought to confirm these results in primary rat Schwann cells. We used the same trafficking assay to quantitate the trafficking of WT and L16P PMP22 in both glycosylated and nonglycosylated (N41Q) forms. As was seen in HEK293 cells, elimination of N-glycosylation led to increased total WT PMP22, whereas total expression of L16P was effectively independent of glycosylation (Fig. 2*A*). For both WT and L16P, elimination of glycosylation resulted in dramatic increases in levels of surface-trafficked protein (Fig. 2*B*). For N41Q PMP22, this was not due solely to its higher total expression levels relative to WT: both N41Q PMP22 and the L16P/N41Q double mutant also exhibited higher surface-trafficking *efficiencies* (Fig. 2*C*). These results recapitulate our HEK293 observations for PMP22 in Schwann cells, the cells most relevant to the physiological role of PMP22 in PNS myelination and CMTD.

These results appear inconsistent with the results of Ryan *et al.*, (39), where the authors explored the role of N-linked glycosylation on WT PMP22 stability, oligomerization, and localization. They observed no differences in PMP22 localization between the glycosylated and nonglycosylated forms using cell surface biotinylation or confocal microscopy. However, the authors of that work noted that they could have missed changes in PMP22 distribution due to limitations in the techniques then available. The trafficking assay employed in this study is both robust and quantitative (24, 27, 44), likely explaining why this change in PMP22 trafficking efficiency owing to N-linked glycosylation was not previously detected.

### Mechanism of PMP22 glycosylation

In light of the observation that N-linked glycosylation limits PMP22 forward trafficking, we sought to uncover the pathway by which this modification occurs. In mammalian cells, the OST complex catalyzes the transfer of a preassembled oligosaccharide from a dolichol pyrophosphate-linked donor onto the target protein (36, 37). Mammalian cells express two OST complexes with different catalytic subunits (STT3A and STT3B), a shared set of noncatalytic subunits, plus some complex-specific subunits (45). Complexes containing STT3A (OST-A) are associated with the Sec61-translocon and catalyze canonical cotranslational glycosylation as proteins are threaded into the membrane (46, 47). Complexes containing STT3B (OST-B) are not associated with the translocon and catalyze glycosylation posttranslationally (47, 48). We sought to uncover which OST complex—OST-A or OST-B—was responsible for mediating PMP22 glycosylation.

**Figure 3:**
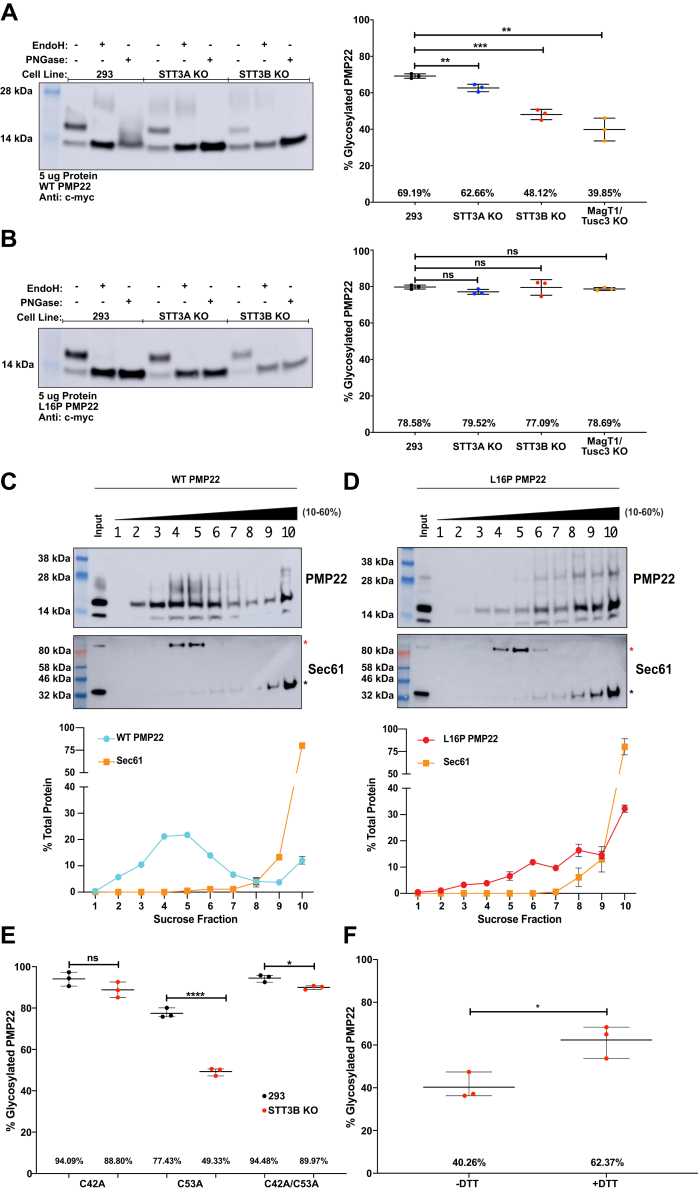
**Mechanism of PMP22 glycosylation**. *A* and *B*, Western blots showing WT PMP22 (*A*) or L16P (*B*) PMP22 glycosylation in HEK293 cells, STT3A KO HEK293 cells, or STT3B HEK293 cells. Glycosidase treatments with EndoH or PNGase were used to confirm the identity of the bands. Quantified levels of glycosylated PMP22 from three independent biological replicates are shown on the right. Uncut Western blots are shown in Figure S3. *C* and *D*, sucrose density fractionation of cell lysates containing WT (*C*) or L16P (*D*) PMP22. Representative blots showing the distribution of PMP22 and Sec61 are shown, with quantified levels of the two proteins from three independent biological replicates shown below. Antibodies directed against Sec61α1 were used to identify Sec61. The *black asterisk* indicates monomeric Sec61α, and the *red asterisk* represents an unidentified (possibly detergent-resistant Sec61α/TRAP complex oligomers) Sec61α-containing complex; both densities were used for quantification. Uncut Western blots are shown in Figure S5. *E*, quantified levels of glycosylated C42A, C53A, or C42A/C53A PMP22 in HEK293 (*black*, see also Fig. S6) or STT3B KO HEK293 (*red*, see also Fig. S7) cells from three independent biological replicates. *F*, quantified levels of glycosylated WT PMP22 in STT3B KO HEK293 cells that were either not treated or treated with 2 mM dithiothreitol (DTT) for 2 h prior to cell lysis. All error bars represent standard deviations and, if not shown, were too small to be visualized. Students *t* test was used for all statistical analyses. ns, not significant, ∗*p* < 0.05, ∗∗*p* < 0.01, ∗∗∗*p* < 0.001, ∗∗∗∗*p* < 0.0001.

We quantified PMP22 glycosylation *via* Western blotting in cell lysates from both HEK293 cells and from cells in which STT3A or STT3B had been genetically knocked out using CRISPR-Cas9 (Fig. 3) (45). WT PMP22 separates into three distinct bands on an SDS-PAGE gel (Figs. 3*A* and S3*A*), where the identities of each can be confirmed *via* comparison with gel patterns following treatment of samples with different glycosidases. The lower band corresponds to nonglycosylated PMP22 (the band is unchanged upon treatment with glycosidases), the middle band corresponds to ER-resident PMP22 (as identified by its disappearance upon treatment with EndoH), and the top smear corresponds to post-ER PMP22 (as identified by its resistance to EndoH and disappearance upon treatment with PNGase). Quantification of the fraction of PMP22 that is glycosylated in these cell lines from three independent biological replicates revealed that 69 ± 2% (mean ± standard deviation) of total WT PMP22 is glycosylated in HEK293 cells, 63 ± 3% is glycosylated in STT3A knockout (KO) cells, and 48 ± 3% is glycosylated in STT3B KO cells (Fig. 3*A*). These results suggest that, although both OST complexes are able to glycosylate WT PMP22, posttranslational glycosylation mediated by OST-B appears to be the predominant pathway, as the STT3B KO cell line caused a significant loss in PMP22 glycosylation, while the level of glycosylation in the STT3A KO cell line remained similar to normal HEK293 cells. To confirm this, we also quantified WT PMP22 glycosylation in MagT1/Tusc3 double KO cell lines. MagT1 and Tusc3 are accessory proteins found exclusively in the OST-B complex (49). In this cell line, 40 ± 7% of WT PMP22 was glycosylated, confirming OST-B as the predominant pathway of N-glycosylation for WT PMP22.

We next quantified the levels of L16P PMP22 glycosylation in these four cell lines (Figs. 3*B* and S3*B*). In HEK293 cells 80 ± 2% of L16P PMP22 was glycosylated, whereas 77 ± 2% was glycosylated in STT3A KO cells, 80 ± 5% was glycosylated in STT3B KO cells, and 79 ± 1% of was glycosylated in MagT1/Tusc3 double-KO cells. This contrasts with the WT PMP22 results, suggesting that L16P PMP22 has no preference for glycosylation *via* OST-A *versus* OST-B. We also quantified the levels of glycosylation in HEK293, STT3A KO, and STT3B KO cell lines for A67T, G93R, and T118M PMP22 (Fig. S4) and found similar results as for L16P PMP22. These results suggest that, unlike with the moderately stable WT protein, less stable and misfolding-prone CMTD variants of PMP22 can be glycosylated equally well co- or posttranslationally.

Why is glycosylation of WT PMP22, but not CMTD variants, sensitive to the loss of OST-B? One hypothesis is that the conformational instability of the CMTD mutants increases the time they remain associated with the translocation machinery and therefore in proximity to OST-A than for WT PMP22. As seen in Figure 3*A*, a significant portion of WT PMP22 is still glycosylated even in the absence of STT3B, suggesting that WT PMP22 is still a substrate, albeit suboptimal for OST-A glycosylation. To clarify the above hypothesis, we monitored direct association of WT and the unstable L16P form of PMP22 with the Sec61 translocon. To this end, we performed sucrose density fractionation of PMP22-containing cell lysates (50). Western blotting was used to locate PMP22 as well as the Sec61α subunit of the translocon (4, 5, 6, 46) in the gradient. As seen in Figure 3*C*, WT PMP22 preferentially partitioned in fractions 3 to 6 with a second minor population partitioning in fraction 10. Sec61α predominantly partitioned in fractions 9 and 10, indicating that the majority of WT PMP22 is not associated with the translocon, as expected for this relatively stable form of the protein. Figure 3*D* shows the fractionation of L16P PMP22. Unlike WT PMP22, L16P PMP22 partitions into a higher-density fraction, where it exhibits significant copartitioning with Sec61α. This suggests that more L16P PMP22 is retained at the translocon, explaining its more efficient glycosylation by STT3A compared with the WT protein.

In Figure 3*C*, lanes 2 and 3 and Figure 3*D*, lanes 3 and 4 (see also Fig. S5) we notice glycosylated PMP22 accumulates in the low-sucrose fractions of the gradient. Since the PM is more cholesterol rich than intracellular membranes, proteins found in this environment would accumulate in the low-density portions of the gradient. We believe that the sucrose density fractionation experiments suggest that a significant fraction of PMP22 at the PM is glycosylated, although this finding was not the purpose of these experiments.

The second question arising from Figure 3*A* is why does a significant fraction of WT PMP22 escape OST-A glycosylation as it is threaded into the ER lumen? Although its relative stability seems to be one factor, we also explored a possible role for the residue cysteine 42. It has been observed that glycosylation sequences with a cysteine residue at the N + 1 position relative to the glycosylation site (-N-C-S/T sequences) tend to be skipped by OST-A (49). Human PMP22 contains a cysteine residue adjacent to the N41 glycosylation site. To test the importance of C42 in PMP22 glycosylation, we made the C42A mutation and measured PMP22 glycosylation in HEK293 cells and in STT3B KO cells. In addition, we mutated the other solvent-exposed cysteine in PMP22 to an alanine (C53A) and also constructed the double cysteine to alanine mutations (C42A/C53A). Figures 3*E* and S6 show that, like WT, the glycosylation of C53A PMP22 is sensitive to the loss of the OST-B complex, leading to a reduction in glycosylation (77 ± 3% glycosylated in HEK293 cells *versus* 49 ± 2% glycosylated in STT3B KO cells). This suggests that C53A PMP22 is still predominately glycosylated posttranslationally *via* OST-B. However, in the single C42A or double C42A/C53A PMP22 mutants, glycosylation was no longer sensitive to STT3B KO. C42A PMP22 was glycosylated 94 ± 4% in HEK293 cells and 89 ± 4% in STT3B KO cells, whereas C42A/C53A PMP22 was glycosylated 95 ± 2% in HEK293 cells and 90 ± 1% in STT3B KO cells. These Figure 3*F* data suggest that these mutants can be glycosylated *via* OST-A. Of interest, C42A and C42A/C53A PMP22 exhibited higher levels of glycosylation in HEK293 cells than WT PMP22, implying that the WT C42 helps make PMP22 a suboptimal substrate for STT3A glycosylation.

Structural studies comparing OST-A and OST-B complexes do not show significant differences in the active sites that could explain the differences in substrate specificity (51). Based on homology to the claudins, there is likely a C42–C53 intramolecular disulfide bond in PMP22 (18). The fact that the C53A mutation did not affect PMP22 glycosylation ruled out the possibility that a disulfide bond between C42 and C53 impedes PMP22 glycosylation by STT3A. However, we hypothesized that the redox state of C42 may be responsible for making PMP22 a suboptimal substrate for OST-A. To test this hypothesis, we measured PMP22 glycosylation in STT3B KO cells that had been treated with small amounts (2 mM) of the reducing agent dithiothreitol (DTT) for 2 h prior to cell lysis (Figs. 3*F* and S7). This short treatment is not long enough to induce changes in the ER proteome by activating the unfolded protein response (52). We observed an increase in PMP22 glycosylation in STT3B KO cells from 40 ± 7% in untreated cells to 62 ± 8% in cells treated with DTT, a significant increase. This result combined with that observed in Figure 3*E* suggests that the efficiency of N-glycosylation of WT PMP22 by OST-A is modulated by the thiol redox potential of the lumen of the ER in a way that is dependent on PMP22 C42, with a more reducing environment resulting in higher efficiency of N-glycosylation by OST-A. This suggests the possible involvement of another ER-resident thiol redox protein perhaps *via* transient disulfide bond formation with C42.

### PMP22 trafficking in response to loss of glycosylation machinery

**Figure 4:**
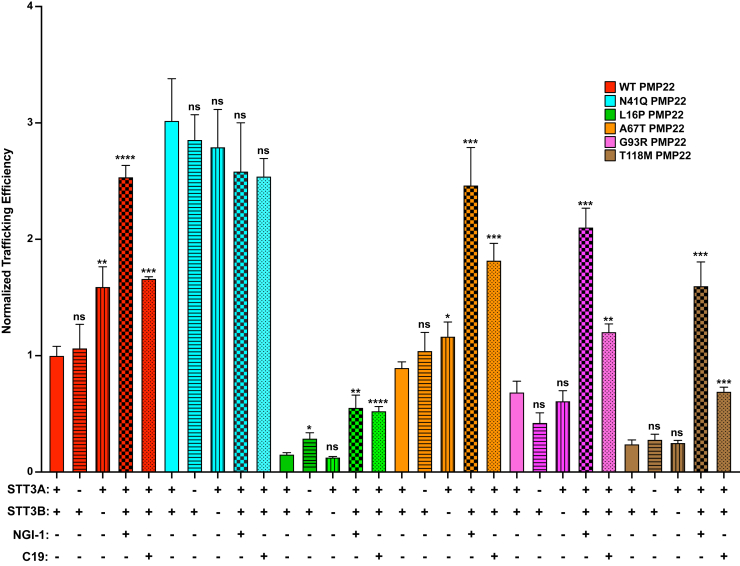
**PMP22 trafficking efficiencies measured in the presence of glycosylation inhibitors.** Normalized trafficking efficiencies of PMP22 variants in HEK293 cells (*solid color*), STT3A KO HEK293 cells (*horizontal stripes*), STT3B KO HEK293 cells (*vertical stripes*), HEK293 cells treated for 24 h with 10 μM NGI-1 (*checkered*, OSTA/OSTB inhibitor), or HEK293 cells treated for 24 h with 10 μM C19 (*dots*, OSTB inhibitor), each from three independent biological replicates. All efficiency values were normalized to WT PMP22 trafficking in untreated HEK293 cells in paired biological replicates. Error bars represent SD. Student’s *t* test was used to compare the trafficking efficiencies in untreated cells with that in KO cell lines or in cell lines treated with the inhibitors. ns, not significant, ∗*p* < 0.05, ∗∗*p* < 0.01, ∗∗∗*p* < 0.001, ∗∗∗∗*p* < 0.0001.

We next tested what happens to PMP22 trafficking when specific mechanisms of glycosylation were inhibited. We measured PMP22 trafficking in STT3A and STT3B KO cell lines (Fig. 4, horizontal and vertical striped graphs, respectively). In addition, we measured PMP22 trafficking under conditions in which both complexes or STT3B were inhibited for 24 h prior to the experiment (Fig. 4 checkered and dotted plots, respectively). NGI-1, a partial inhibitor of both STT3A and STT3B (53), resulted in decreased WT PMP22 glycosylation to 12 ± 1% (Fig. S8). C19, a specific inhibitor of STT3B (54), reduced WT PMP22 glycosylation to 48 ± 2%, similar to what was observed in the STT3B KO cells (Fig. S8).

In Figure 4 we measured PMP22 trafficking efficiencies under these various conditions. All efficiency values were normalized relative to WT PMP22 in paired experiments using either untreated HEK293 cells (experiments with STT3A or STT3B KO cells) or HEK293 cells treated with dimethyl sulfoxide (experiments with NGI-1 or C19). As expected, STT3A KO had no effect on WT PMP22 trafficking efficiency, confirming its minor role in mediating WT PMP22 glycosylation. However, in STT3B KO cells WT PMP22 exhibits a 1.6 ± 0.4-fold increase (mean ± standard deviation) in trafficking efficiency. Treatment of HEK293 cells with NGI-1 further increased WT PMP22 trafficking efficiency 2.5 ± 0.2-fold over untreated cells, similar to the trafficking efficiency of N41Q PMP22. Treating cells with the STT3B-specific inhibitor C19 caused a 1.7 ± 0.1-fold increase over untreated cells. These results confirm that OST-B is the major facilitator of WT PMP22 glycosylation.

Since N41Q PMP22 is not glycosylated, we expected no changes in its trafficking efficiency under any of these OST inhibitory conditions. As predicted, none of these experimental conditions significantly altered the trafficking efficiency of N41Q PMP22 (Fig. 4). This result serves as an internal control for our experiments by showing that neither use of OST KO cell lines nor pharmacological inhibition of OST caused glycosylation-independent global changes for this mutant in its protein folding quality control and surface trafficking efficiency.

Inhibition of both OSTs *via* NGI-1 caused increases in trafficking efficiency for all CMTD PMP22 variants studied (Fig. 4). However, neither STT3A nor STT3B KO cell lines dramatically altered the trafficking efficiencies of these variants. This is consistent with the data in Figures 3*B* and S4 showing that KO of either STT3A or STT3B did not reduce the glycosylation levels of these PMP22 variants to the same extent as for WT PMP22. Of interest, specific inhibition of STT3B with C19 caused an increase in PMP22 trafficking efficiencies of all three CMTD mutants. Our data clearly indicate a significant fraction of PMP22 is glycosylated posttranslationally by STT3B and that inhibition of this process, either genetically or pharmacologically, increases PMP22 trafficking efficiency.

### Identification of PMP22 interactor proteins

**Figure 5:**
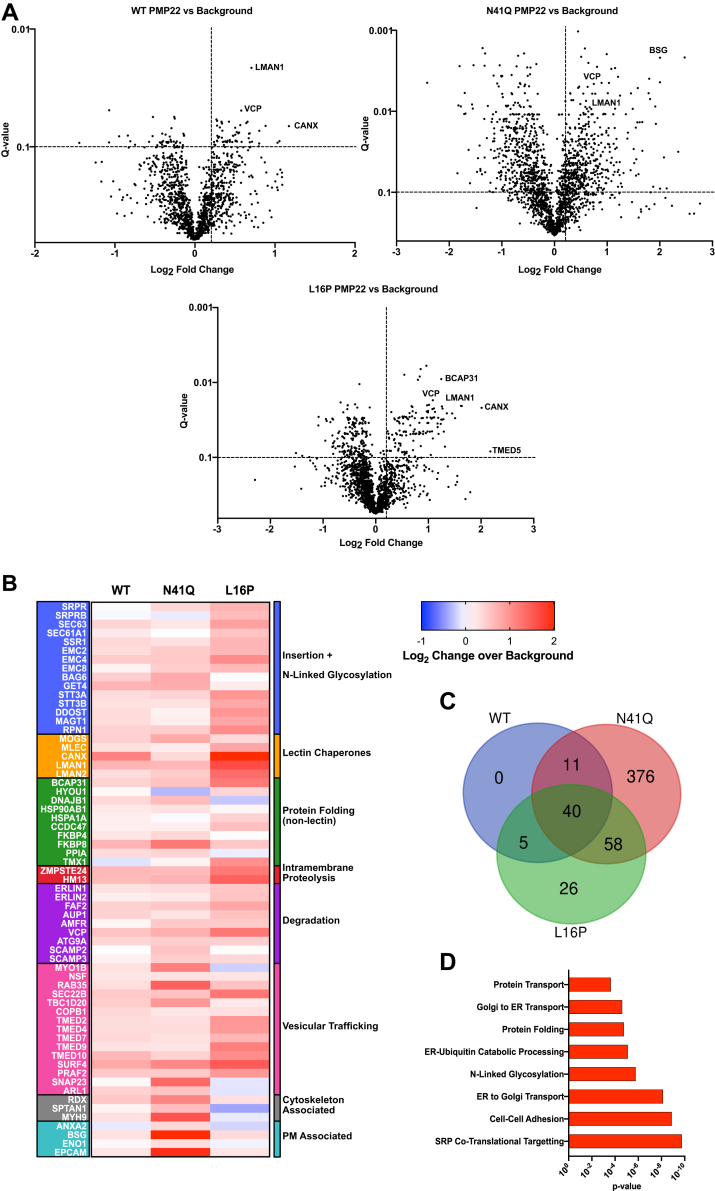
**Proteomics to uncover novel PMP22-interacting partners.** Coimmunoprecipitation and LC-MS/MS proteomics was used to identify proteins that interact with PMP22. *A*, Volcano plots for WT, N41Q, and L16P PMP22 showing interacting proteins that were identified from six biological replicates. Log_2_-fold change of the identified protein in the WT PMP22 sample as compared with samples obtained from IPs with untagged PMP22 are shown on the *x*-axis, and the statistical significance (Q-value) for the interactions from the multiple replicates is shown on the *y*-axis. Selected interactions are identified in which dotted lines separate the data into quadrants with the upper right-hand quadrant signifying PMP22 interactors. *B*, heat map showing quantified interactions for WT, N41Q, or L16P PMP22 over background. The heat map is arranged temporally in regard to PMP22 biogenesis. The top of the map identifies interactions predicted to occur early in biogenesis, and the bottom of the map shows interactions predicted to occur later in biogenesis. All interactions had a Log_2_-fold change over background of >0.2 and a Q-value of <0.1 and were identified in multiple biological replicates for at least one PMP22 variant. *C*, Venn diagram showing overlap of filtered interaction partners identified for WT, N41Q, or L16P with a Log_2_-fold change over background >0.2, Q-value < 0.1. *D*, pathway enrichment analysis showing the GO_Biological Processes enrichment from the identified proteins that interacted with at least one of the three forms of PMP22 probed (WT, N41Q, or L16P). A Fisher Exact *p*-value test was used to calculate the *p*-values for the GO_term enrichment.

We next sought to uncover proteins responsible for limiting or promoting PMP22 trafficking. Previous work has established that WT PMP22 interacts with the ERQC proteins CNX and RER1 (31, 32, 33, 35). In order to discover novel PMP22 interactors, we turned to coimmunoprecipitation (co-IP) and liquid chromatography–mass spectrometry/mass spectrometry (LC-MS/MS)-based proteomics. We expressed myc-tagged WT and mutant forms of PMP22 in HEK293 cells, immunopurified the protein using magnetic beads conjugated to myc antibodies under gentle lysis conditions, and used shotgun proteomics to identify proteins that immunopurified with PMP22. Tandem mass tag (TMT) labeling was used to quantitatively compare interactions across multiple samples (55, 56). We compared interactions with WT PMP22 *versus* N41Q PMP22 to uncover glycosylation-specific interactions. Furthermore, WT *versus* L16P PMP22 measurements were used to uncover interactions that depend on protein stability. Also included were purifications from a mock lysate (cells expressing untagged PMP22) to identify nonspecific background proteins. Overall, six biological co-IP replicates of mock-transfected cells, WT, N41Q, and L16P PMP22 were included and distributed across four 6-plex TMT mass spectrometry batches (Table S1). Figure 5*A* displays examples of the PMP22 interacting proteins identified by our screen. We compared the TMT intensities of identified proteins in copurifications of the three forms of PMP22 relative to the mock background to provide a list of filtered interaction partners. We defined PMP22 interactors identified in multiple biological replicates to be those proteins that exhibit a Storey Q-value < 0.1 and log_2_ fold change >0.2 compared with mock samples (Table S1, Fig. 5*A*).

Overall, we identified 56 proteins that appear to interact either directly or indirectly with WT PMP22 (Fig. 5, *B* and *C*, Table S1). In addition, 485 and 129 proteins appear to interact with N41Q and L16P PMP22, respectively, with 40 interactors being seen to interact with all three forms of the protein (Fig. 5, *B* and *C*). It may seem surprising that N41Q and L16P displayed such a large number of unique filtered interactors that failed to pass the defined interactor Q-value cutoff for WT. However, this is most likely due to batch-to-batch variability in the MS analysis, which led to more statistical power in identifying interactions for N41Q and L16P compared with WT (Fig. 5*A*, Table S1). Despite these variable protein identifications, it is important to note that the TMT labeling ensures that a quantitative signal is present for all samples allowing for direct comparisons of partner protein abundances across the different PMP22 variants, even if an interactor was only filtered as enriched for the mutant PMP22.

We then used the DAVID bioinformatic database to initially group these interactions based on gene ontology (GO) terms followed by a manual curation of the interactions based on literature analysis to compose the diagram shown in Figure 5*B* to visualize interactions known to be involved in protein biogenesis, quality control, or other possible functions (57, 58). Here the TMT quantitative signal allows for direct comparison of protein enrichment. The heatmap is organized temporally with respect to the start of biogenesis at the translocon, with interactions predicted to occur earlier in PMP22 biogenesis shown at the top (insertion and N-linked glycosylation) and those expected to occur later (plasma membrane localized) shown at the bottom. We also carried out an unbiased pathway enrichment analysis using the identified interactors for WT, N41Q, and/or L16P PMP22. Figure 5*D* shows biological processes that were the most highly represented, confirming an overrepresentation of processes related to protein trafficking and ERQC.

Not surprisingly, all three forms of PMP22 probed are seen to interact directly or indirectly with proteins playing roles at all phases of the translocon-to-plasma membrane secretory network, including chaperones and proteins associated with degradative shunts (Fig. 5, *B* and *D*). It is noteworthy that PMP22 is seen to interact both with cytoskeletal proteins (Fig. 5*B*) and proteins involved in cell–cell adhesion (Fig. 5*D*), consistent with previous studies (15, 59, 60, 61, 62, 63).

WT, glycosylation-deficient N41Q, and unstable L16P mutant forms of PMP22 also exhibited a number of *differences* in their protein interactive profiles. Relative to WT and L16P, N41Q was seen to be especially prone to interact with proteins late in the secretory pathway and at the cell surface (Fig. 5*B*). This makes sense in light of the results presented in Figure 1 that N41Q PMP22 trafficks to the cell surface much more efficiently than WT PMP22. On the other hand, the unstable L16P shows a much higher enrichment for interactions associated with proteins at the stages of “insertion and N-linked glycosylation” and “lectin chaperone.” This suggests that more avid binding events occur early on for this variant. Intriguingly, L16P PMP22 appears to interact more strongly with components of the recently discovered ER membrane protein complex (EMC) than WT or N41Q PMP22 (Fig. 5*B*). The EMC has been shown to be involved in early folding events and associated QC of multispan membrane proteins of low hydrophobicity (64, 65). This suggests that the EMC may be playing a role in PMP22 maturation, a link begging address in future studies. It was gratifying to see that both the Sec61α translocon component and STT3A associate more avidly with the unstable L16P PMP22 mutant relative to WT protein, consistent with the biochemical studies of this paper. It was also reassuring that we were able to identify CNX in our screens and that the order of affinity of CNX for different forms of PMP22 was L16P>WT>N41Q, as supported by previous literature results (31, 32, 33, 34).

### Clarification of possible roles for selected chaperones in PMP22 trafficking

**Figure 6:**
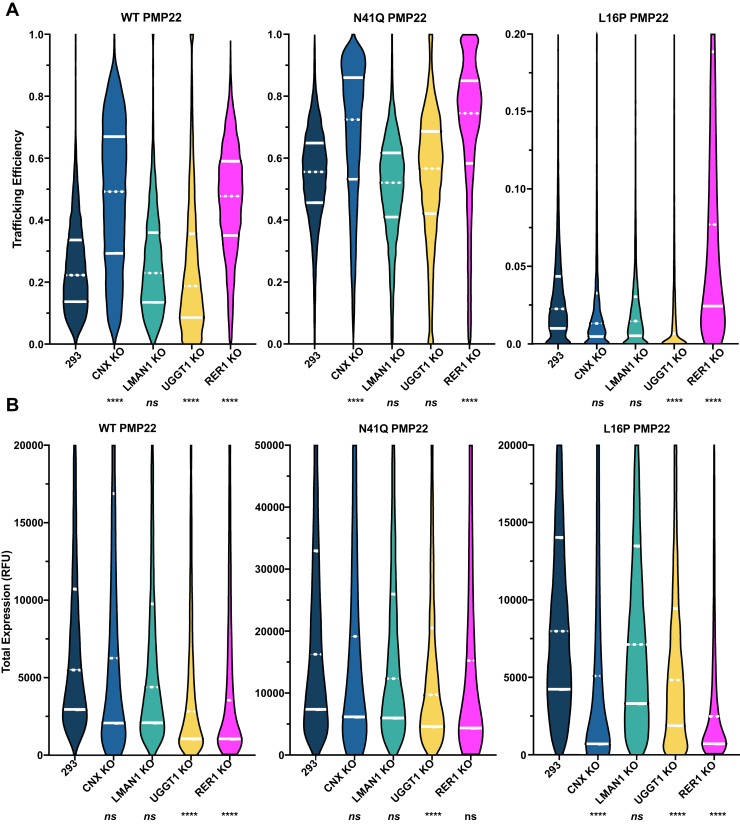
**PMP22 trafficking efficiencies in CRISPR-Cas9 KO cells of potential ERQC interactors.** CRISPR-Cas9 was used to generate KO cells of selected potential proteins involved in mediating trafficking effects for PMP22 (Fig. S10). Violin plots of population distributions of WT, N41Q, and L16P PMP22 trafficking efficiencies from three biological replicates are shown. Data were collected in HEK293 cells (*navy*), CNX KO HEK293 cells (*blue*), LMAN1 KO HEK293 cells (*green*), UGGT1 KO HEK293 cells (*yellow*), and RER1 HEK293 KO cells (*pink*). *White lines* in the population distributions separate the data into quartiles, whereas the dashed line in each case represents the median. The pairwise Kolmogorov–Smirnov test was used to compare population distributions in KO cells to 293 cells, ∗∗∗∗*p* < 0.0001 and ns, not significant.

We set out to validate the role of selected interactors from Figure 5*B* in mediating PMP22 trafficking. We also were interested in the non-lectin chaperone RER1 in light of a previous report that it is involved in ERQC for PMP22 (35). To validate whether these proteins do indeed function in PMP22 trafficking, we generated CRISPR-Cas9 clonal KO cell lines (Fig. S9) and quantitated PMP22 trafficking efficiency therein. We focused on four potential mediators of PMP22 trafficking: CNX, lectin mannose-binding protein 1 (LMAN1, also known as ER–Golgi intermediate compartment 53 kDa protein—ERGIC53), UDP-glucose:glycoprotein glucosyltransferase 1 (UGGT1), and RER1. CNX is believed to retain PMP22 in the ER to promote folding (31, 32, 33, 34, 35), whereas RER1 is a sorting receptor in the early Golgi and has previously been shown to function in retrograde trafficking of two PMP22 variants, L16P and G150D (35). LMAN1 was one of the strongest interactors observed in our screen (Fig. 5, *A* and *B* and Table S1) and is a mannose-specific lectin that has previously been shown to promote maturation of glycoproteins from the ER to the Golgi complex (66, 67, 68). We hypothesized that LMAN1 might be responsible for promoting maturation of PMP22 in a glycan-specific manner. UGGT1 is a main component of the CNX cycle and has been proposed to act as its main folding sensor (69, 70, 71). We did not identify UGGT1 in our screen, suggesting short lifetimes for UGGT1–PMP22 complexes. However, since PMP22 was seen to interact strongly with CNX and since UGGT1 is a main component of the CNX cycle we hypothesized a likely role for it in PMP22 maturation. UGGT1 functions by reglucosylating folding-immature client proteins to cause reengagement with CNX or by recognizing polypeptides as folded, resulting in a “no glucosylate” decision then being made that allows the client protein to forward-traffic beyond the ER (10, 72). We hypothesized that elimination of UGGT1 would cause an increase in forward trafficking of PMP22 in a glycosylation-dependent manner. Figure 6*A* shows trafficking efficiencies of WT, N41Q, and L16P PMP22 in the four KO cell lines compared with HEK293 cells. Also shown is the total cellular level for each form of the protein (Fig. 6*B*). The data are presented as violin plots, which show the population distribution of total cellular PMP22 and surface trafficking efficiencies in individual cells from three independent biological replicates measuring >2000 cells per replicate. The horizontal white lines separate the data into quartiles. Data for A67T, G93R, and T118M PMP22 are presented in Figure S10.

Knockout of CNX caused a dramatic increase in PMP22 surface trafficking efficiency for both WT and the glycosylation-deficient N41Q PMP22 (Fig. 6*A*). Relative to parental HEK293, the increase for WT is from a median trafficking efficiency of 22% to 49%. For N41Q the increase in median trafficking efficiency was from 56% to 72%. The fact that this increase was independent of glycosylation was not surprising in light of previous experiments showing that, even though CNX is a “lectin chaperone” it can engage PMP22 independent of its glycosylation state (34). These results are at variance with previous studies in which confocal microscopy was used to show that CNX knockdown did not change the distribution of GFP-tagged PMP22 in cells (35). This discrepancy may be explained by use in the previous study of GFP-tagged PMP22, which is prone to aggregate owing to the propensity of GFP to oligomerize (73). Our results suggest that the modest (ca. 20%) trafficking efficiency by which WT PMP22 surface-trafficks partly reflects intracellular retention by CNX, an activity that CNX evidently carries out using both glycosylation-dependent and -independent mechanisms. Such CNX-specific intracellular retention was not seen to be accompanied by significantly changed total cellular levels of WT and N41Q PMP22 (Fig. 6*B*) as might have been expected if retention was linked to degradation.

In contrast to the results for WT, L16P PMP22 showed a modest reduction in trafficking efficiency when CNX was knocked out (Fig. 6*A*). This indicates CNX is not the primary determinant of forward trafficking for L16P. However, knockout of CNX did result in a roughly 4-fold reduction in the total cellular L16P PMP22 level (Fig. 6*B*). This is consistent both with the data of Figure 5*B* and with previous data that CNX does recognize and form long-lived complexes with L16P PMP22 (31, 34).

It was also seen that G93R and T118M, which have stabilities intermediate between WT and L16P (27) both exhibit modest trafficking enhancement in CNX KO cells (Fig. S10*A*). On the other hand, A67T, which has WT-like stability, exhibits a dramatic (WT-like) enhancement of surface trafficking (Fig. S10*A*). These results again point to CNX as a main determinant of PMP22 surface trafficking efficiency for WT and moderately stable mutants, but not for the most unstable disease mutant forms. All three of these mutants were L16P-like in that they also exhibited a significant reduction in total cellular levels in CNX KO cells (Fig. S10*B*), suggesting CNX may also help protect the cellular levels of these forms of PMP22.

Despite the fact that LMAN1 appears to form avid complexes with WT, N41Q, and L16P PMP22 (Fig. 5*B*), knockout of LMAN1 showed little effect on the trafficking efficiency of any of the PMP22 variants under study (Figs. 6*A* and S10*A*). This result may imply functional redundancy of other ER sorting receptors, such as LMAN2, with LMAN1 (see Fig. 5*B*). This interpretation will need to be tested.

UGGT1 KO cells showed a significant decrease in PMP22 trafficking efficiency for WT, L16P, G93R, and T118M but not for glycosylation-deficient N41Q PMP22 (Figs. 6*A* and S10*A*). It is not surprising that N41Q PMP22 exhibited no changes in trafficking efficiency in UGGT1 KO cells since this PMP22 variant does not contain an N-glycan moiety for UGGT1 to modify. By reglucosylating the PMP22 N-glycan, UGGT1 promotes reassociation with calnexin and retention of the immature protein within the ER so that it has additional time to complete folding. In this regard, it is also notable that a significant decrease was seen in total cellular WT and all mutant PMP22 levels except for N41Q in UGGT1 KO cells (Fig. 6*B*). Overall, these results point to an overall glycosylation-dependent pro-folding and expression level protective role of UGGT1 for PMP22.

RER1 KO cells showed increases in trafficking efficiencies for WT and all PMP22 variants under study (Figs. 6*A* and S10*A*). This confirms previous results indicating a role for RER1 in ERQC of PMP22 (35). The present results suggest that RER1 functions as a glycosylation-independent Golgi-to-ER retrograde transporter for both WT and disease mutant forms of PMP22, a function that reflects a role for RER1 in preventing the escape of folding-immature proteins from the ER. Total cellular levels of WT and of all mutants with the possible exception of N41Q were also reduced in RER1 KO cells (Figs. 6*B* and S10*B*), suggesting that RER1 also protects various forms of PMP22 against degradation.

## Discussion

### N-glycosylation limits forward trafficking and usually lowers total cellular levels of PMP22

Both here and in previous work WT PMP22 was seen to traffic to the PM with modest efficiency (24, 26, 27, 28). Disease mutant forms of the protein usually traffic with even lower efficiency, in some cases approaching 0% (27, 42). A key result of this work is that elimination of the single N-glycosylation site in PMP22 (N41) results in several-fold increases in the surface trafficking efficiencies of both WT and disease mutant forms of the protein (Fig. 1). While N-glycosylation is often associated with promoting molecular recognition, protein stability, or protein solubility (10, 36, 37, 38), here it is seen to play a limiting role in PMP22 trafficking from the ER to the PM. This reflects the role of protein N-glycan moieties in ERQC, where they serve as a “quality control barcode” to which monosaccharides are added or removed to signal to the ERQC machinery the folding status of the nascent protein, forming the basis for determining whether that protein will be allowed to forward-traffic beyond the ER, be retained in the ER for further attempts at folding, or whether it should be targeted for degradation (36, 37, 70, 71). Mutations of N41 have yet to be discovered among the documented CMTD mutant forms of PMP22. Consistent with its role in ERQC of PMP22, previous studies have shown that N-glycosylation is not important for the ability of PMP22 to partition into cholesterol-rich membrane domains (40), form specific *trans* interactions with other myelin proteins (74), or modulate lipid ultrastructure *in vitro* (19).

Even unstable and poorly trafficking disease mutant forms of PMP22 exhibit a dramatic enhancement in surface trafficking efficiency upon elimination or inhibition of N-glycosylation. While the well-justified question should be posed as to whether these disease mutants are functional even if they do traffic to the plasma membrane, it is nevertheless interesting to wonder if inhibition of glycosylation for these mutant forms of PMP22 could lead to partial restoration of Schwann cell function to alleviate disease progression and symptoms in patients with CMTD. Testing this idea will require extensive additional investigation. Inhibition of glycosylation to increase trafficking efficiency could also be investigated as a possible therapeutic approach for the hereditary neuropathy with liability to pressure palsies form of CMTD (∼20% of all CMTD cases (17, 20)), which is caused by loss of one WT PMP22 allele (haploinsufficient WT/null conditions). Partial inhibition of N-glycosylation has emerged as a potential therapeutic approach for treating certain forms of cancer (53, 54). These treatments may be worthy of investigation for treatment of certain forms of CMTD. However, the fact that mutations in STT3A and STT3B, the catalytic subunits to OST-A and OST-B, respectively, are linked to congenital disorders of glycosylation (75) provides a cautionary note.

In addition to enhancing surface trafficking efficiency, elimination of glycosylation was also seen to significantly increase the total levels of WT and moderately stable forms of PMP22 in cells. The most likely explanation for this is that elimination of glycosylation reduces the amount of PMP22 that is degraded. It should also be emphasized that not all the nondegraded fraction of PMP22 trafficks to the cell surface; much of the protein is retained intracellularly, particularly for the disease mutant forms. Previous studies have suggested that nondegraded misfolded PMP22 accumulates in the ER-to-Golgi intermediate compartment (ERGIC) (42, 76, 77, 78). Future study will be required to confirm the location of this mistrafficked-but-not-degraded PMP22 and whether it is cytotoxic.

It is important to note that the very unstable L16P disease mutant form differs from WT and moderately stable disease mutant forms in that it does not appear to be rescued from degradation when it is not glycosylated. This suggests that most of its degradation is not *via* the classical ERAD pathway but occurs prior to engagement of this mutant with the degradation-targeting elements of that pathway. Our proteomics results (Fig. 5*B*) may offer clues regarding the identity of these pathways based on the observed preferential association of the intramembrane proteases ZMPSTE24, and HM13 with L16P.

### Different glycosylation pathways for WT and CMTD variants of PMP22

N-linked glycosylation is mediated by the OST complexes either cotranslationally *via* OST-A or posttranslationally *via* OST-B (45, 47). Our results show that, for WT PMP22, a significant fraction of the nascent protein is glycosylated by OST-B. In contrast, the severely folding-defective L16P disease mutant form of PMP22 is glycosylated to a much higher extent by OST-A. Indeed, this mutant cofractionates with the Sec61 translocon. The prolonged residency of L16P PMP22 at the translocon appears to enable glycosylation of PMP22 by OST-A before it reaches OST-B. The L16P mutation site is located in the first transmembrane helix of the protein, where it has been shown by NMR spectroscopy to induce a folding-destabilizing kink in this helix that causes it to dissociate from the other three transmembrane helices (43). The structural and folding defects induced by this mutation are evidently manifest to quality control even while the downstream protein sequence is still being translated, providing an example of misfolding that occurs at the stage of membrane integration (27, 79).

### Clarification of the roles of CNX, UGGT1, and RER1 in ERQC management of PMP22 folding and trafficking

CNX has previously been reported to monitor the folding status of PMP22 in a manner that can be either glycosylation dependent or independent (31, 32, 33, 34, 35). Fontanini *et al*. (34) showed that CNX can bind both WT and L16P in their glycosylated forms, with the L16P complex being much longer lived than WT. That same study showed that N41Q PMP22 does not avidly bind CNX, whereas the double L16P/N41Q mutant does, a result that is echoed by our proteomics data. (Fig. 5*B*) Here, it was observed that the surface-trafficking of WT and N41Q are both dramatically increased upon CRIPSR knockout of CNX. CNX is clearly a major gate keeper for the forward trafficking of WT PMP22, whether or not it is glycosylated. It is not yet clear whether CNX can act alone on PMP22 or act in complex with additional ERQC factors (69, 80). CNX was also seen to play a role in retaining moderately unstable disease mutant forms of PMP22 (Fig. S10). Of interest, although CNX does not limit the surface trafficking of L16P, it does appear to help protect it from degradation (Fig. 6).

UGGT1 is believed to monitor the folding status of glycoproteins upon nascent entry into the ER and also after they have been released from CNX (10, 11, 37, 69, 70, 71). UGGT glucosylates the N-glycoside of proteins deemed not to have completed folding, resulting in their association with CNX and retention in the ER for repeated attempts to complete folding. Here, we found KO of UGGT1 in HEK293 cells caused a decrease in the trafficking efficiency for both WT and most disease variant forms but did not change the trafficking efficiency of N41Q PMP22. It was also seen that there is a decrease in total WT PMP22 levels in UGGT1 KO cells compared with parental HEK293 cells. The results were consistent with the classical model for UGGT1 action. The N-glycan-dependent engagement of PMP22 by UGGT1 both promotes its folding in the ER by keeping PMP22 in the CNX cycle and may also help to protect it from degradation *via* ERAD.

Finally, we explored the effect of RER1 KO on PMP22 trafficking efficiency. RER1 had previously been characterized to play a role in retrieving misfolded L16P and G150D PMP22 disease mutants from the Golgi and returning these proteins to the ER (35). It thereby appears to play a role in recognizing misfolded PMP22 that escapes the quality control network, resulting in retrotrafficking back to the ER for further attempts at folding. RER1 KO caused an increase in the surface trafficking efficiency of L16P PMP22. We also found that both WT and N41Q PMP22 exhibited increased trafficking efficiency in the RER1 KO cell line. This is not surprising in light of data showing that WT PMP22 is itself only a marginally stable protein and surface trafficks with low efficiency.

## Conclusions

This work highlights N-glycosylation of PMP22 as a factor that very significantly reduces the surface trafficking efficiency of this tetraspan membrane protein. It was seen that the molecular pathways of N-glycosylation are different for WT PMP22 and less stable forms. The results also clarified the roles of CNX and RER1 in PMP22 trafficking and showed that UGGT1 appears to play role in quality control of PMP22 folding/trafficking, whereas LMAN1 appears not to be important. Finally, this work provides a proteomic census of PMP22-interactive proteins that includes a readout of their differential levels of association with WT relative to both L16P and to glycosylation-deficient N41Q PMP22. This work therefore illuminates ERQC of PMP22 folding and trafficking while also providing information that will hopefully be useful in guiding future efforts to complete a map of the complex ERQC landscape experienced by PMP22 under conditions of both health and disease.

## Experimental methods

For a list of antibodies and guide RNAs (gRNAs) used in this study please see Table S2. NGI-1 was purchased from Sigma-Aldrich and C19 (N-(5-(Morpholinosulfonyl)-2-(pyrrolidin-1-yl)phenyl)benzamide) was purchased from Mcule.

### Cloning

Human cDNA for PMP22 was subcloned into a pIRES2 mammalian expression vector containing a green fluorescent protein (GFP) downstream of the IRES site. This allowed both PMP22 and GFP to be expressed as independent proteins from the same expression vector. To make PMP22 immunologically detectable we used QuikChange mutagenesis to insert a myc epitope into the second extracellular loop of PMP22 within the pIRES2 vector. QuikChange mutagenesis was also used to make the various point mutations used in this study. Plasmids were purified using a GenElute HP Plasmid MidiPrep Kit (Sigma-Aldrich). To generate plasmids for CRISPR-Cas9 KO cell lines, DNA encoding single guide RNAs was subcloned into pSpCas9-2A-Puro V2.0 (Addgene).

### Cell culture and transfection

HEK293 cells were obtained from the American Type Culture Center; STT3A KO, STT3B KO, and MagT1/Tusc3 DKO HEK293 cells were a gift from Dr Reid Gilmore. Cells were cultured in Dulbecco’s modified Eagle medium (DMEM) containing 10% fetal bovine serum (FBS) and 1% pen/strep at 37 °C and 5% CO_2_. HEK293 cells were transiently transfected using the calcium phosphate method. Plates of size 6 cm^2^ were transfected with 1.5 μg plasmid DNA. Plates of size 10 cm^2^ were transfected with 5 μg plasmid DNA. Transfections were consistently >50% efficient.

Rat Schwann cells (RSCs) were a gift from the Carter laboratory. RSCs were cultured in high-glucose DMEM with L-glutamine and sodium pyruvate containing 10% FBS, 1% penicillin/streptomycin, and 2 μM forskolin at 37 °C and 5% CO_2_. RSCs were transiently transfected with lipofectamine 3000. Cells were grown in 6-cm dishes and transfected with 2.5 μg plasmid DNA.

### Single-cell trafficking assay

Single-cell flow cytometry was performed as previously described (24, 27). Briefly, ∼36 h post transfection HEK293 cells were trypsinized and prepared for flow cytometry analysis using the Fix & Perm kit in accordance with the manufacturer instructions (Life Technologies). Cells were suspended in 100 μl of culture media, and a PE-labeled monoclonal anti-myc antibody was added to the solution to a final concentration of 0.75 μg/ml to immunostain surface PMP22. Cells were then incubated in the dark for 30 min at room temperature. Fixation solution, 100 μl, was then added to the media, and the cells were incubated in the dark for 15 min. The cells were then rinsed with 3 ml wash buffer (phosphate-buffered saline, PBS, containing 5% FBS and 0.1% NaN_3_) and pelleted *via* centrifugation twice. The cells were then suspended in 100 μl of permeabilization solution and incubated with an Alexa Fluor 647–labeled monoclonal anti-myc antibody at a final concentration of 0.75 μg/ml to label intracellular PMP22. Following 30 min of incubation in the dark, the cells were again rinsed with 3 ml wash buffer and pelleted *via* centrifugation twice. Cell were then resuspended in 300 μl wash buffer prior to flow cytometry analysis.

Immunostained cells were analyzed with a FACS Canto II flow cytometer (BD Bioscience). Single cells were selected based on their light scattering area and width profiles. A total of 2500 transfected cells expressing PMP22 were analyzed from each sample by gating on GFP-positive cells (excited with a 488-nm laser, detected with 515–545 nm emission filter). The single-cell PE intensity (surface PMP22, excited with a 488-nm laser, detected with 564–606 nm emission filter) and Alexa Fluor 647 intensity (internal PMP22, excited with a 633-nm laser, detected with 650–670 nm emission filter) signals were corrected for nonspecific binding by subtracting the average intensities of untransfected, GFP-negative cells within each sample. To correct for the difference in the fluorescence intensity of the two antibodies, cells expressing WT PMP22 were stained with either the PE-labeled antibody or the Alexa Fluor 647–labeled antibody prior to flow cytometry analysis, and the ratio of the average intensities of these cells was used to normalize the two signals. Single-cell trafficking efficiency values were then calculated from the ratio of the corrected PE signal of a given cell over the sum of its corrected Alexa Fluor 647 and PE signals. Average trafficking efficiency values calculated in this fashion were found to be similar to those determined by a comparison of the population-averaged intensities of intact (surface PMP22) and permeabilized (total PMP22) cells stained with the same concentration of the same fluorescently labeled antibody. Single-cell fluorescence intensity values below the background intensity were assigned an intensity of 0. Results were analyzed and visualized using FlowJo X software (Treestar Inc).

From titrations of both intact and permeable cells expressing WT PMP22 with fluorescently labeled antibodies, we found the average fluorescence intensity to be linearly dependent on the antibody concentration. This confirms that fluorescence intensity values fall within the linear range of the detectors. Moreover, this ensures that the observed trafficking efficiency values are independent of the chosen antibody concentration. Compensation for spillover of the fluorescence signals between the channels utilized for the analysis as well as the gates for the selection of single cells, GFP-positive cells, and GFP-negative cells was initially set manually but was kept consistent for the collection of all data sets obtained thereafter.

Single-cell trafficking for myc-tagged PMP22 in Schwann cells was determined through the same method as described above for HEK293 cells, with three replicates and 250 to 900 EGFP-positive cells analyzed per replicate (the number of cells corresponds to as many cells as possible in each replicate).

### Co-IP and sample preparation for mass spectrometry

Approximately 36 h post transfection, confluent 10 cm^2^ plates were harvested *via* scraping in ice-cold PBS and pelleted *via* centrifugation. Cells were then lysed in lysis buffer (40 mM Hepes, 100 mM NaCl, 2 mM EDTA, 0.3% CHAPS, 10% glycerol, 1 mM PMSF, 1× HALT protease inhibitor, pH 7.8) for 20 min at 4 °C with gentle rotation. Insoluble fractions were then removed *via* centrifugation, and protein concentration was determined *via* Bradford assay. One hundred and fifty micrograms of total protein lysate was then mixed with 15 μl of anti-myc magnetic beads (Pierce) preequilibrated with lysis buffer and the volume brought up to 500 μl with TBS (25 mM Tris, 100 mM NaCl, pH 7.8). The mixture was then mixed with end-over-end rotation at 4 °C for 90 min. Beads were then washed with 3× 250 μl volumes of TBS plus 0.25% Tween-20. Samples were then eluted with 2× 50 μl washes of elution buffer (50 mM Tris, 300 mM NaCl, 4% SDS, 2 mM TCEP, 1 mM EDTA, pH 7.8) with beads vortexed and allowed to equilibrate with elution buffer for 15 min at 37 °C for each wash.

Following elution, proteins were precipitated using chloroform/methanol extraction, and protein pellets were allowed to air dry for 60 min at room temperature. Protein pellets were then resuspended in 50 μl 0.1% RapiGest SF (Waters). Disulfide bonds were reduced with 1 mM TCEP and free sulfhydryl groups acetylated with 1 mM iodoacetamide, and samples were incubated for 30 min at room temperature in the dark. Samples were then digested with 0.5 μg trypsin overnight at 37 °C under 700 rpm shaking. Samples were then labeled using 6-plex TMTs (Thermo Scientific) according to the manufacturers protocol. TMT-labeled samples were then mixed and acidified with formic acid to a pH < 2. The volume of the samples was then reduced to one-sixth the initial volume on a SpeedVac and then adjusted back to the original volume with Buffer A (H_2_O, 5% acetonitrile, 0.1% formic acid) to precipitate and remove RapiGest SF.

### Liquid chromatography–tandem mass spectrometry

MudPIT microcolumns were prepared as previously described (55, 81). Peptide samples were directly loaded onto the columns using a high-pressure chamber. Samples were then desalted for 30 min with buffer A (97% water, 2.9% acetonitrile, 0.1% formic acid v/v/v). LC-MS/MS analysis was performed using a Q-Exactive HF (Thermo Fisher) or Exploris480 (Thermo Fisher) mass spectrometer equipped with an Ultimate3000 RSLCnano system (Thermo Fisher). MudPIT experiments were performed with 10 μl sequential injections of 0%, 10%, 30%, 60%, and 100% buffer C (500 mM ammonium acetate in buffer A), followed by a final injection of 90% buffer C with 10% buffer B (99.9% acetonitrile, 0.1% formic acid v/v) and each step followed by a 130-min gradient from 5% to 80% B with a flow rate of 300 nl/minute when using the Q-Exactive HF and 500 nl/minute when using the Exploris480 on a 20-cm fused silica microcapillary column (ID 100 um) ending with a laser-pulled tip filled with Aqua C18, 3 μm, 100-Å resin (Phenomenex). Electrospray ionization (*ESI*) was performed directly from the analytical column by applying a voltage of 2.0 kV when using the Q-Exactive HF and 2.2 kV when using the Exploris480 with an inlet capillary temperature of 275 °C. Using the Q-Exactive HF, data-dependent acquisition of mass spectra was carried out by performing a full scan from 300 to 1800 m/z with a resolution of 60,000. The top 15 peaks for each full scan were fragmented by higher-energy collisional dissociation using normalized collision energy of 38, 0.7 m/z isolation window, 120 ms maximum injection time, at a resolution of 15,000 scanned from 100 to 1800 m/z and dynamic exclusion set to 60 s. Using the Exploris480, data-dependent acquisition of mass spectra was carried out by performing a full scan from 400 to 1600 m/z at a resolution of 120,000. Top-speed data acquisition was used for acquiring MS/MS spectra using a cycle time of 3 s, with a normalized collision energy of 36, 0.4 m/z isolation window, 120 ms maximum injection time, at a resolution of 30,000 with the first m/z starting at 110. Peptide identification and TMT-based protein quantification was carried out using Proteome Discoverer 2.3 or 2.4. MS/MS spectra were extracted from Thermo Xcalibur .raw file format and searched using SEQUEST against a UNIPROT human proteome database (released 03/2014 and containing 20,337 entries). The database was curated to remove redundant protein and splice isoforms and supplemented with common biological MS contaminants. Searches were carried out using a decoy database of reversed peptide sequences and the following parameters: 10 ppm peptide precursor tolerance, 0.02 Da fragment mass tolerance, minimum peptide length of six amino acids, trypsin cleavage with a maximum of two missed cleavages, dynamic methionine modification of 15.995 Da (oxidation), static cysteine modification of 57.0215 Da (carbamidomethylation), and static N-terminal and lysine modifications of 229.1629 Da (TMT 6-plex). SEQUEST search results were filtered using Percolator to minimize the peptide false discovery rate to 1% and a minimum of two peptides per protein identification. TMT reporter ion intensities were quantified using the Reporter Ion Quantification processing node in Proteome Discoverer 2.3 or 2.4 and 576 summed for peptides belonging to the same protein.

The final mass spectrometry data reflected the result of six biological replicates each of Mock, WT, N41Q, and L16P constructs. Data were acquired across four separate MudPIT experiments.

### Interactome characterization and pathway enrichment analysis

To identify true interactors from nonspecific background TMT intensities first underwent a log_2_ transformation and were then median normalized using the formula:

$$I_{n,TMT\alpha }^{norm}=I_{n,TMT\alpha }^{unnorm}•\frac{\sum_{TMT_{\gamma }}^{TMT_{\alpha }}M}{M_{TMT\alpha }}$$

where $I_{n,TMT\alpha }^{norm}$ and $I_{n,TMT\alpha }^{unnorm}$ are the normalized and unnormalized TMT intensities for a given protein found in TMT channels α-γ. TMT ratios were then calculated by subtracting the log_2_-transformed and median-corrected TMT intensity of the mock channels from the PMP22 channels. The mean log_2_ interaction differences were then calculated across multiple biological replicates. Significance of interaction differences were calculated using a paired, parametric, two-tailed *t* test and multiple testing correction *via* a false discovery rate estimation (82). Volcano plots to highlight the filtered interactors for individual PMP22 variants (WT, N41Q, and L16P) compared with mock are shown in Figure 5*A*. Overlap of filtered interactors is shown in the Venn diagram in Figure 5*C*. Pathway enrichment analysis was performed on the inclusive list of all filtered interactors using the DAVID bioinformatic server (57, 58).

To build the heat map shown in Figure 5*B*, peptide identifications were filtered to include those that were only identified in at least three biological replicates, had a log_2_ fold interaction greater than 0.2 and had a Q-value < 0.1 compared with mock samples. Identified proteins that passed these criteria were grouped *via* GO_term using the DAVID bioinformatic tool (57, 58). Manual curation of the GO term analysis was then used to build the heatmap in a temporal manner based on literature precedent. GraphPad Prism was then used to generate the heat map to visualize the grouped interactions.

### Glycosylation mapping

For glycosylation mapping experiments cells were harvested and lysed as described above except for 60 min at 4 °C. Following lysis, 5-μg samples of total lysate were treated with no enzyme, EndoH, or PNGase (New England Biolabs) according to the manufacturers protocol for 2 h at 37 °C. Samples were then analyzed *via* Western blotting.

### Sucrose density fractionation

Cells were harvested and lysed as described in glycosylation mapping. Following lysis, 100 μg of cell lysate was layered on top of a 0% to 60% step-wise sucrose density gradient in 1-ml ultracentrifuge tube. Lysates were then centrifuged at 200,000*g* in a Beckman TLA-120.2 rotor for 4 h at 4 °C. Layers of the gradient were then sequentially removed in 100-μl fractions to be analyzed *via* Western blotting.

### Generation of KO cell lines

CRISPR technology was utilized to generate genetic KO cells as previously described (83). gRNAs were designed to target early exons of CNX, Rer1, UGGT1, and LMAN1. gRNA oligomers (gRNA sequences shown in Table S2) were annealed, phosphorylated, and ligated into digested pspCas9(BB)-2A-puro plasmid (plasmid no. 62988, Addgene). HEK293 cells were suspended in 2 ml of DMEM supplemented with 10% FBS and plated in six-well plates. The following day, 5 μg of each plasmid was combined with 10 μl Lipofectamine 2000 reagent (Invitrogen) in 1 ml Opti-MEM and incubated at room temperature for 30 min. The culture medium was replaced with the appropriate plasmid-lipofectamine solution, and cells were incubated at 37 °C for 24 h. The medium was then replaced with fresh DMEM plus 10% FBS, and cells were allowed to recover for 24 h at 37 °C prior to the addition of 0.75 μg/ml puromycin. Cells were incubated at 37 °C for 48 h before replacing the medium. After recovering for ∼1 to 2 weeks, cells were pelleted, resuspended in sorting buffer (PBS + 4% FBS), and strained to separate clumps of cells. Solutions were sorted by flow cytometry using a five-laser BD LSRII with a 100-μm nozzle at the Vanderbilt Medical Center Flow Cytometry Core to isolate single-cell cultures in 96-well plates for each cell line. Clones were incubated until they reached ∼70% confluency and then passaged until enough cells could be harvested for KO validation *via* Western blotting.

### Statistics

Student’s *t* test was used to compare mean values in trafficking experiments and glycosylation mapping experiments. Storey Q-values were used to compare identifications in the proteomics data. Pairwise Kolmogorov–Smirnov test was used to compare population distributions. Statistical analyses were performed in GraphPad Prism and Microsoft Excel.

## Data availability

Proteomic data were deposited to the ProteomeXchange Consortium *via* the PRIDE partner repository with the identifier PXD023091. All other data are either presented in the article, are in the supporting information, or are available from the corresponding author upon request.

## Supporting information

This article contains supporting information.

## Conflict of interest

The authors declare that they have no conflicts of interest with the contents of this article.
