# Supplementary material for: Glycosylation limits forward trafficking of the tetraspan membrane protein PMP22
Source: J Biol Chem. 2021 Apr 30;296:100719. doi: 10.1016/j.jbc.2021.100719 (PMC8191293; doi:10.1016/j.jbc.2021.100719)
Supplement: Figures S1 to S10 [file mmc1.pdf]

Supporting Information for:

Glycosylation Limits Forward Trafficking of the Tetraspan Membrane Protein PMP22

Justin T. Marinko[1,2], Madison T. Wright[3], Jonathan P. Schlebach[4], Katherine R. Clowes[1,2], Darren R. Heintzman[5], Lars Plate[3,6], Charles R. Sanders[1,2]*

[1] Department of Biochemistry, Vanderbilt University, Nashville, Tennessee 37240, United States.

[2]Center for Structural Biology, Vanderbilt University, Nashville, Tennessee 37240, United States.

[3]Department of Chemistry, Vanderbilt University, Nashville, Tennessee 37240, United States.

[4] Department of Chemistry, Indiana University, Bloomington, Indiana, 47405, United States

[5]Department of Pathology, Microbiology, and Immunology, Vanderbilt University, Nashville, Tennessee 37240, United States.

[6]Department of Biological Sciences, Vanderbilt University, Nashville, Tennessee 37240, United States.

[*] **Corresponding Author:** Charles R. Sanders, chuck.sanders@vanderbilt.edu

This PDF includes:

Supporting Figures S1-S10

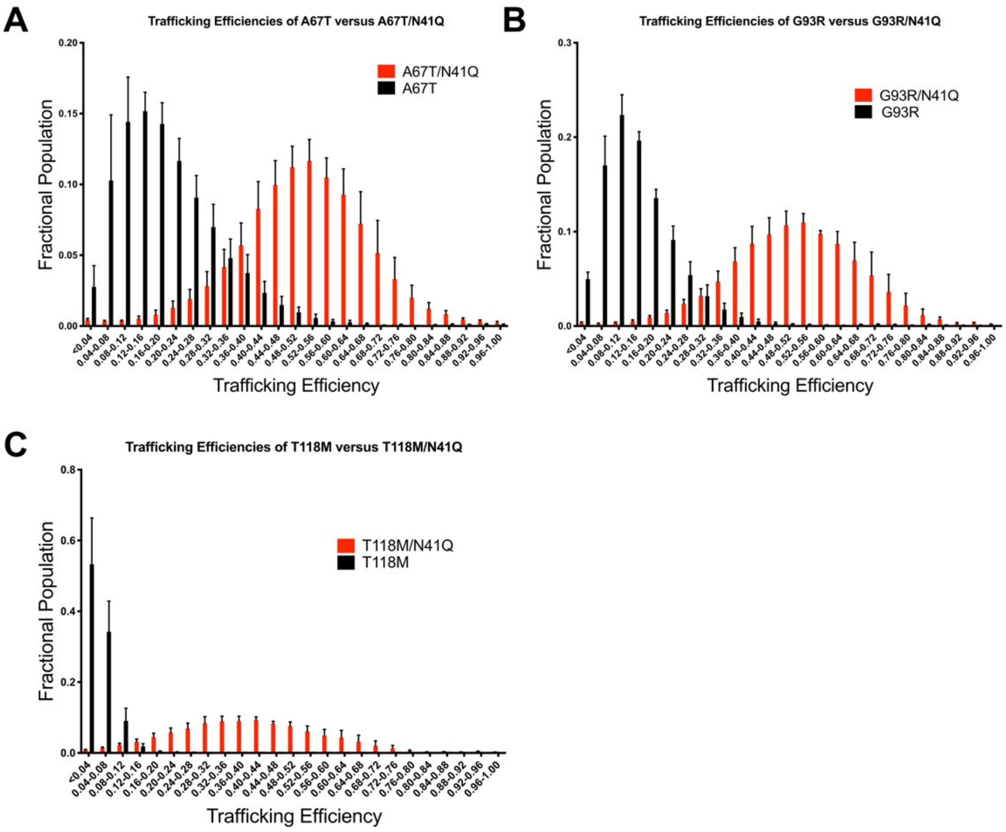

Figure S1. Trafficking efficiencies of CMTD PMP22 variants and their glycosylation deficient parallels**.** Population distribution of PMP22 trafficking efficiencies measured in individual HEK293 cells for (**A**) A67T, (**B**) G93R, and (**C**) T118M PMP22. Values are shown for glycosylated (black) and non-glycosylated (red) variants. Measurements were obtained from 5 biological replicates with 2500 cells measured per replicate. Error bars represent standard deviations (SD) of the replicates.

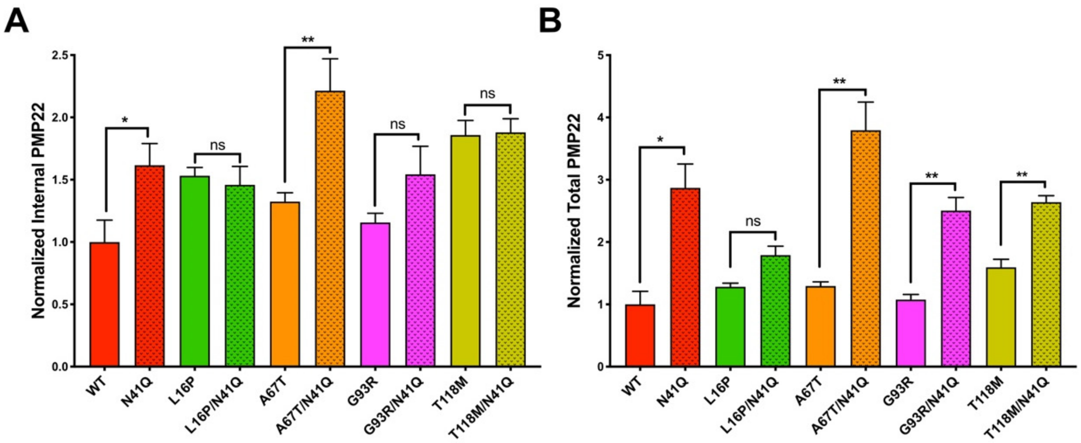

Figure S2. Normalized internal and total PMP22 concentrations. Normalized **(A)** internal and **(B)** total expression of PMP22 variants and their glycosylation deficient partner. Values were obtained from 5 biological replicates with 2500 cells measured per replicate. All values were normalized to WT PMP22 cell surface expression data collected in paired biological replicates. Error bars represent SD of the replicates. Student's t-test was used for statistical analysis. ns=not significant, *=$p<0.05$, **=$p<0.01$.

A

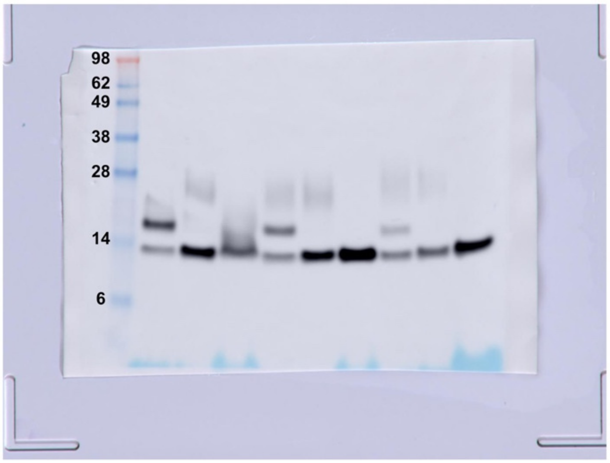

B

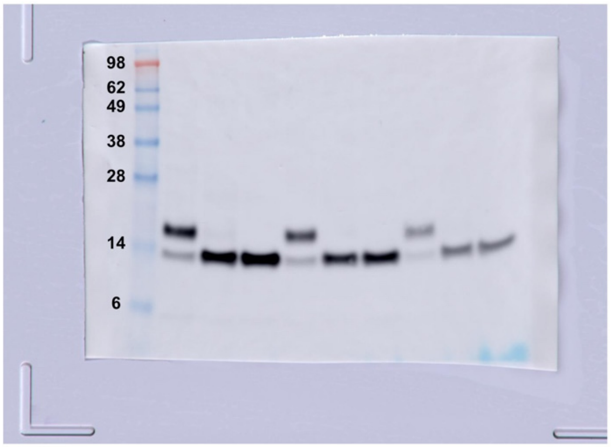

Figure S3. Uncut Western blots from **(A) Figure 3A** and **(B) Figure 3B.**

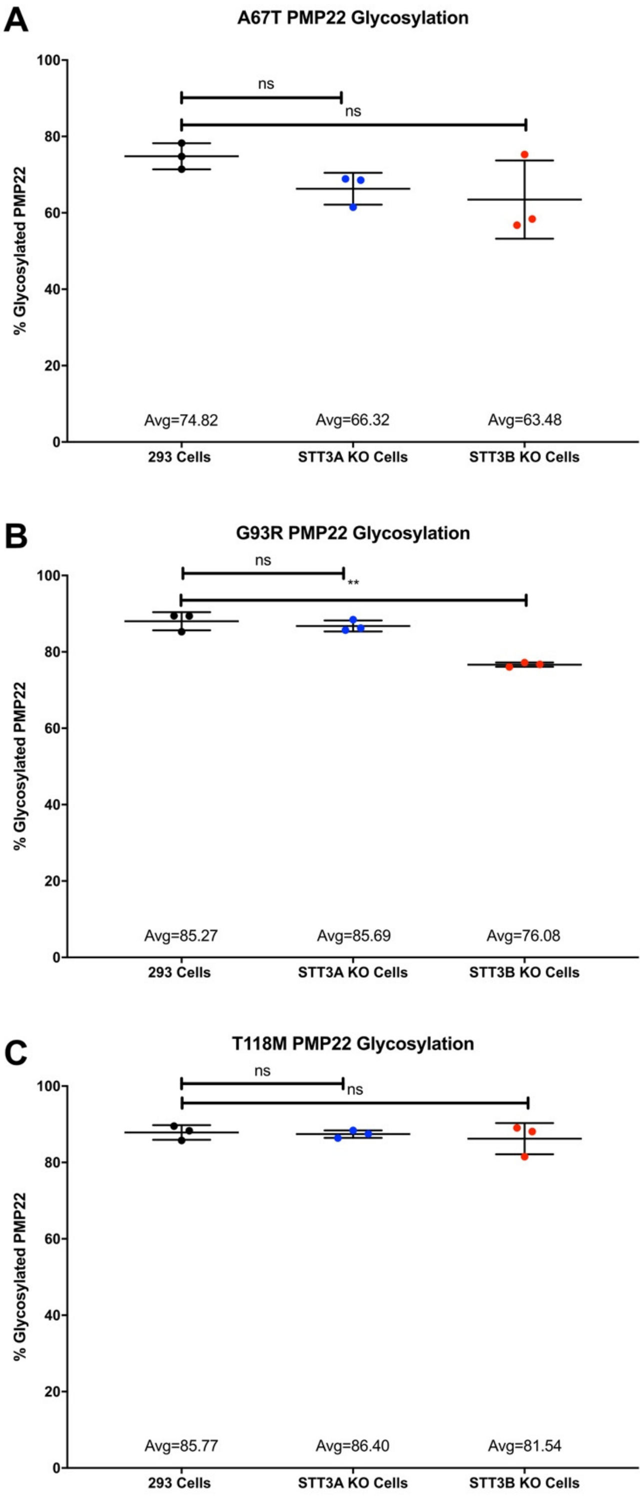

Figure S4. Glycosylation mapping for CMTD PMP22 variants. Quantified levels of glycosylated **(A)** A67T, **(B)** G93R, and **(C)** T118M PMP22 from 3 independent biological replicates.

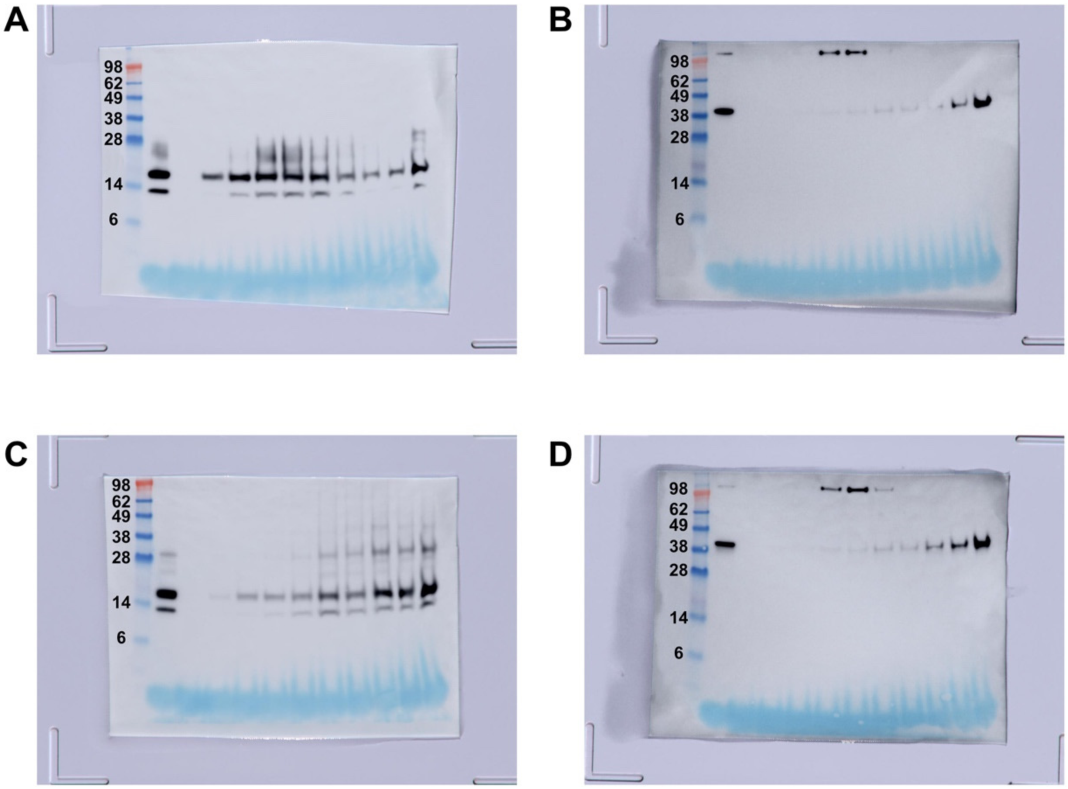

Figure S5. Uncut Western blots from **(A-B) Figure 3C** and **(C-D) Figure 3D**.

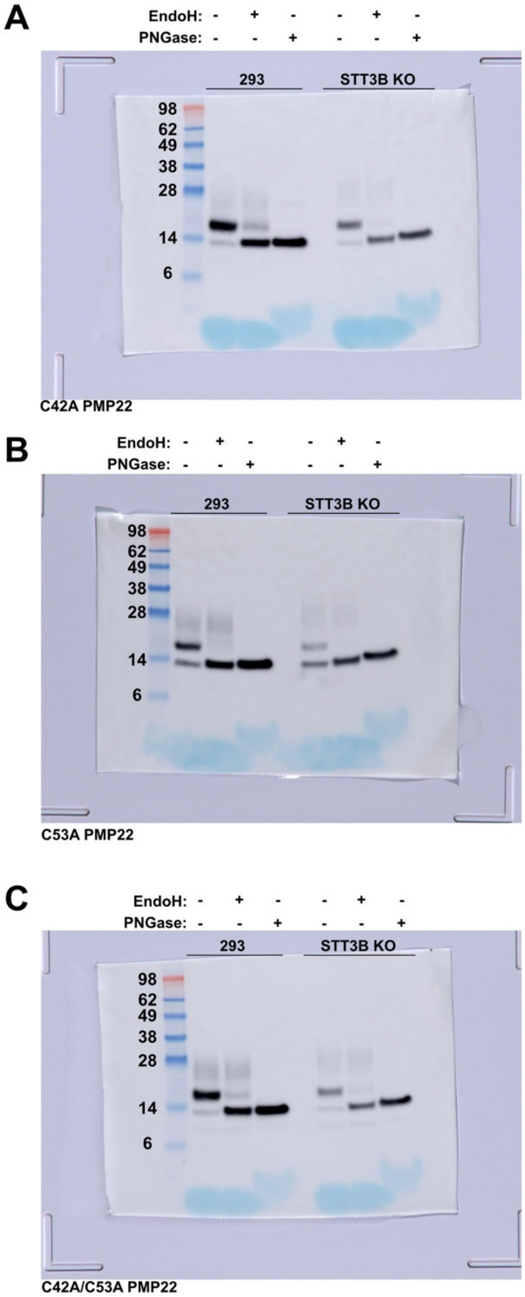

Figure S6. Western blots showing the levels of glycosylation of **(A)** C42A, **(B)** C53A, and **(C)** C42A/C53A PMP22 in HEK293 or STT3B KO HEK293 cells.

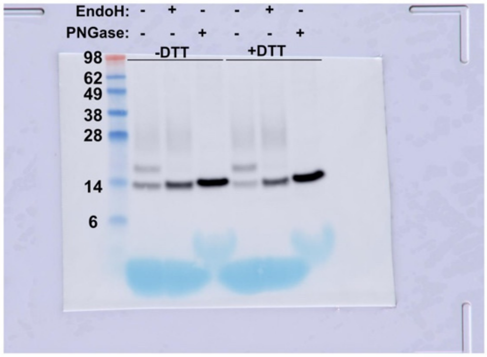

Figure S7. Western blot showing the level of WT PMP22 glycosylation in STT3B KO HEK293 cells that have been pretreated for 2 hours with or without 2 mM DTT.

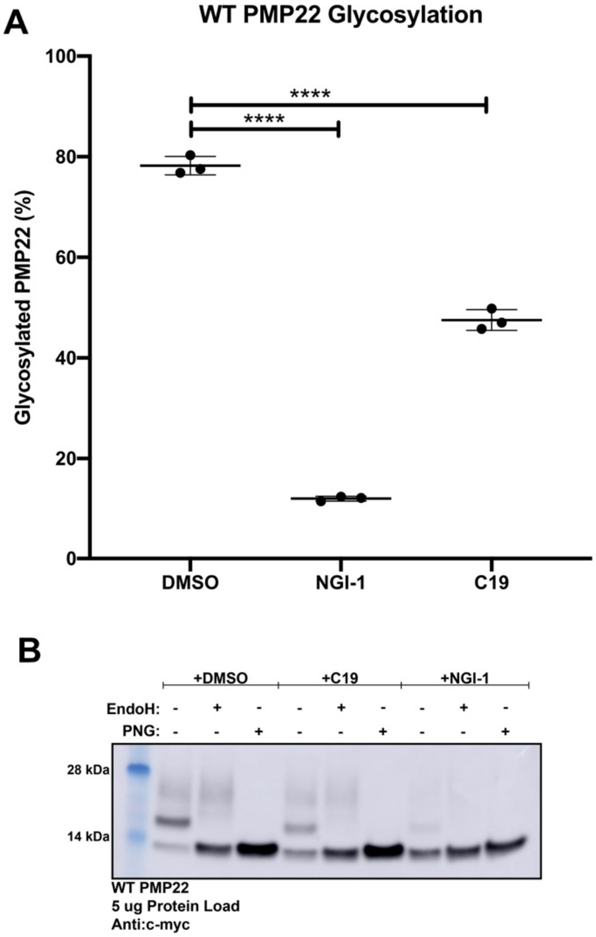

Figure S8. WT PMP22 glycosylation with OST inhibitors. **(A)** Quantification of the levels of WT PMP22 glycosylation from three biological replicates for lysates obtained from cells that had been treated for 24 hours with either DMSO, 10 μM NGI-1, or 10 μM C19. Error bars represent standard deviation. **(B)** Representative

Western blot showing the levels of WT PMP22 glycosylation from lysates obtained from cells that had been treated for 24 hours with either DMSO, 10 μM NGI-1, or 10 μM C19. ****=p<0.0001 using student's t-test.

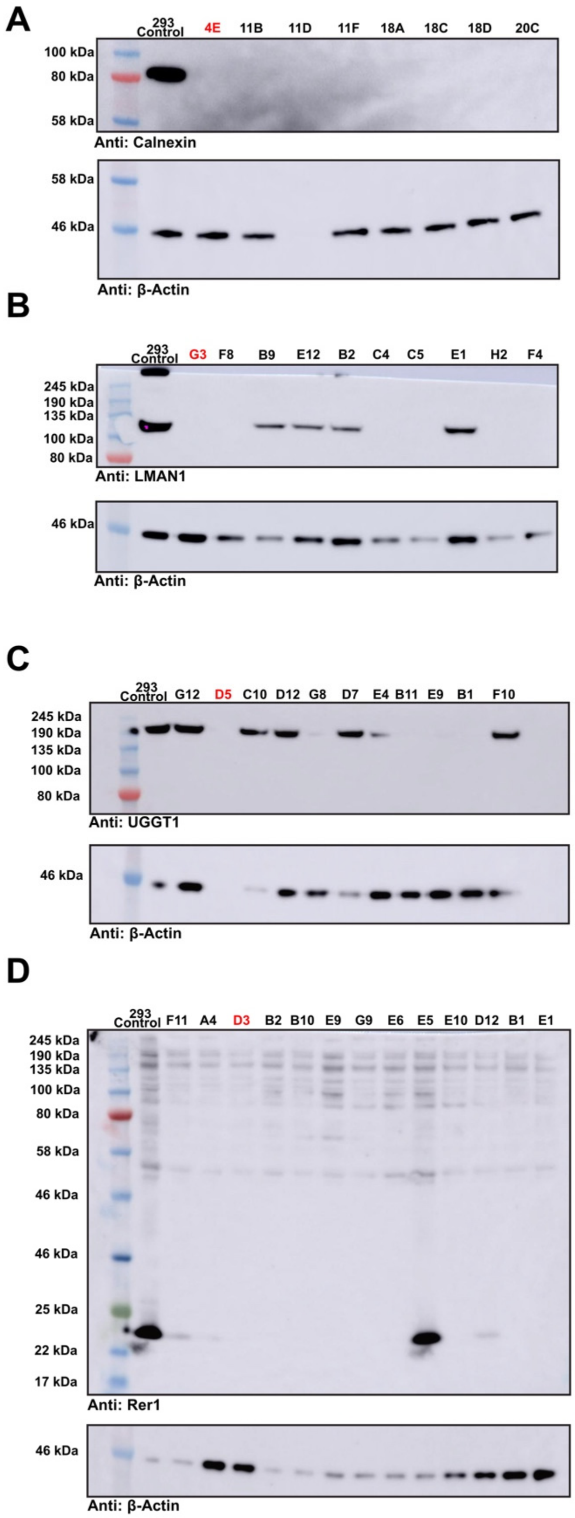

Figure S9. Western blots of CRISPR/Cas9 clonal KO cell lines for **(A)** calnexin (CXN), **(B)** LMAN1, **(C)** UGGT1, and **(D)** RER1. β-Actin was used as a loading control for all samples. Clones selected for trafficking studies are highlighted in red.

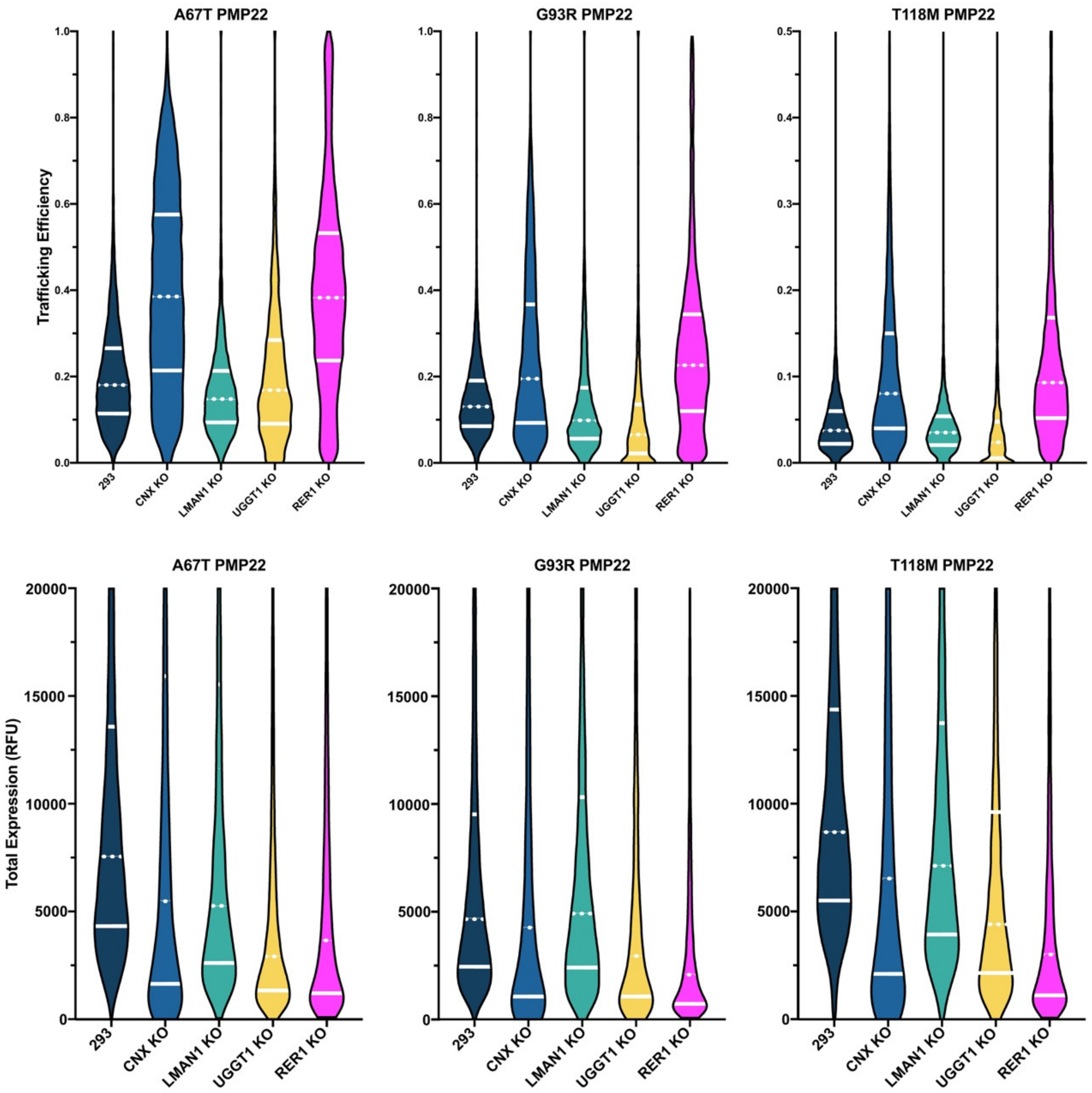

Figure S10. Trafficking assay results for A67T, G93R, and T118M mutant form of PMP22 in various KO cells. **(A)** PMP22 trafficking efficiencies in CRISPR/Cas9 KO cells of potential ERQC interactors. CRISPR/Cas9 was used to generated KO cells of potential proteins involved in mediating trafficking effects for PMP22. Violin plots showing population distributions of A67T, G93R and T118M PMP22 trafficking efficiencies from three biological replicates are shown. Data was collected in HEK293 cells (navy), CNX KO HEK293 cells (blue), LMAN1 KO HEK293 cells (green), UGGT1 KO HEK293 cells (yellow) and RER1 HEK293 KO cells (pink). White lines in the population distributions separate the data into quartiles. **(B)** PMP22 total expression in KO cells. Violin plots showing population distributions of WT PMP22 total, cell surface, or internal expression levels from three biological replicates are shown. Data was collected in HEK293 cells (navy), or UGGT1 KO HEK293 cells (yellow). White lines in the population distributions separate the data into quartiles, with the dotted line representing the median.
